# Refined Structures of *O*-Phospho-l-serine and Its Calcium Salt by New Multinuclear Solid-State NMR Crystallography Methods

**DOI:** 10.1021/acs.jpcb.1c05587

**Published:** 2021-09-23

**Authors:** Renny Mathew, Baltzar Stevensson, Mattias Edén

**Affiliations:** Department of Materials and Environmental Chemistry, Stockholm University, SE-106 91 Stockholm, Sweden

## Abstract

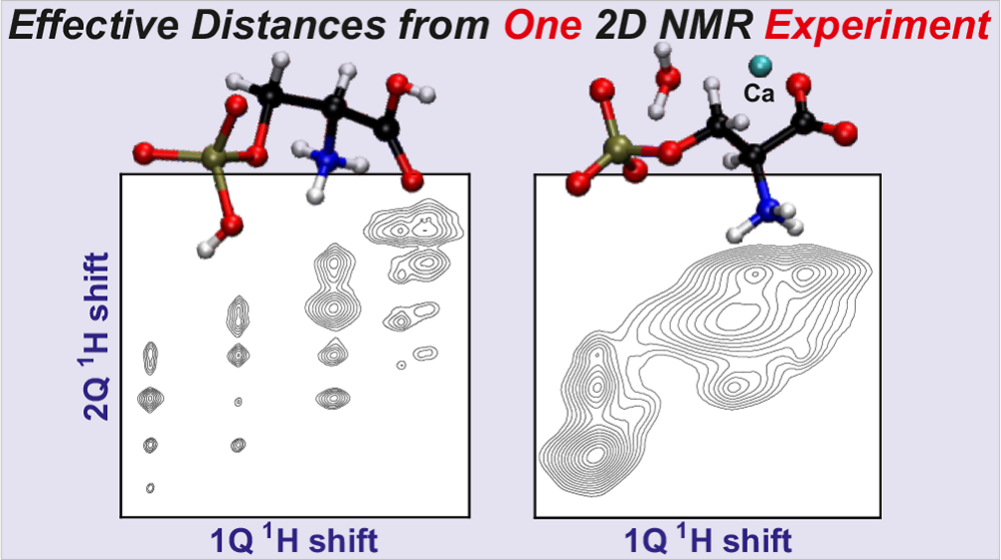

*O*-phospho-l-serine (Pser) and its Ca salt, Ca[*O*-phospho-l-serine]·H_2_O (CaPser), play important roles for bone mineralization and were recently also proposed to account for the markedly improved bone-adhesive properties of Pser-doped calcium phosphate-based cements for biomedical implants. However, the hitherto few proposed structural models of Pser and CaPser were obtained by X-ray diffraction, thereby leaving the proton positions poorly defined. Herein, we refine the Pser and CaPser structures by density functional theory (DFT) calculations and contrast them with direct interatomic-distance constraints from two-dimensional (2D) nuclear magnetic resonance (NMR) correlation experimentation at fast magic-angle spinning (MAS), encompassing double-quantum–single-quantum (2Q–1Q) ^1^H NMR along with heteronuclear ^13^C{^1^H} and ^31^P{^1^H} correlation NMR experiments. The Pser and CaPser structures before and after refinements by DFT were validated against sets of NMR-derived effective ^1^H–^1^H, ^1^H–^31^P, and ^1^H–^13^C distances, which confirmed the improved accuracy of the refined structures. Each distance set was derived from one sole 2D NMR experiment applied to a powder without isotopic enrichment. The distances were extracted without invoking numerical spin-dynamics simulations or approximate phenomenological models. We highlight the advantages and limitations of the new distance-extraction procedure. Isotropic ^1^H, ^13^C, and ^31^P chemical shifts obtained by DFT calculations using the gauge including projector augmented wave (GIPAW) method agreed very well with the experimental results. We discuss the isotropic and anisotropic ^13^C and ^31^P chemical-shift parameters in relation to the previous literature, where most data on CaPser are reported herein for the first time.

## 1 Introduction

**Figure 1:**
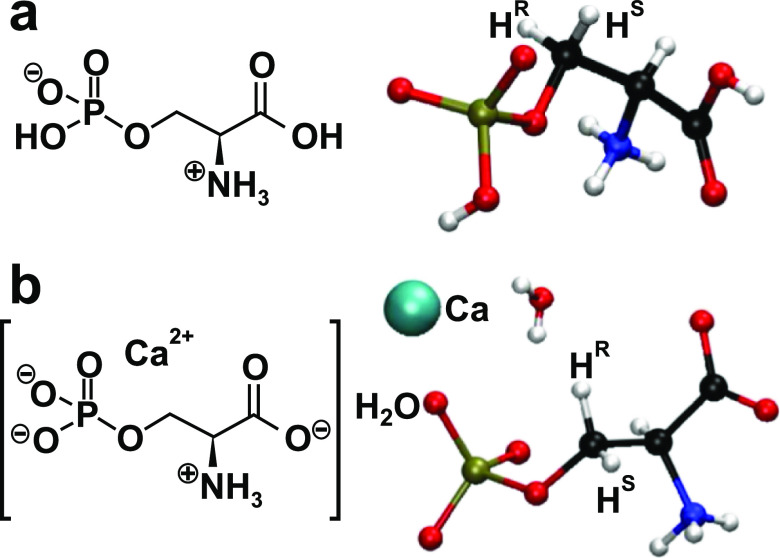
Molecular structure of (a) *O*-phospho-l-serine, and (b) Ca[*O*-phospho-l-serine]·H_2_O shown in the left panel, along with their respective ball-and-stick 3D representations displayed in the right panel according to the X-ray diffraction-derived structures of refs (26) and (34). Note the two crystallographically inequivalent methylene protons that are distinguished by using the stereochemical “R” and “S” nomenclature.^126^

Protein residues with negatively charged side chains stemming from either phosphorylation or carboxy groups occur frequently in many non-collagenous proteins (NCPs) believed to govern the growth of bone and tooth mineral.^1−3^ Bone mineral consists of a carbonated form of the mineral calcium hydroxyapatite (HA), which associates with fibrils of type I collagen to build the hierarchical bone structure.^2−6^ However, the mechanisms behind bone-mineral formation and how they are initiated and controlled remain heavily debated over decades.^2,3,6^ Inarguably, the negatively charged COO^–^ and/or PO_4_^2–^-bearing residues of NCPs render them readily adsorbed at inorganic calcium phosphate (CaP) surfaces,^1−3,7,8^ encompassing bone mineral, HA, and other crystalline as well as structurally disordered CaP phases. One example is the complexes between amorphous calcium phosphate (ACP)^6,9^ and casein in milk.^10−13^ Strong affinities for binding at CaP surfaces are also manifested by small and negatively charged biomolecules, such as amino acids and the ester of l-serine and phosphoric acid, *O*-phospho-l-serine (Pser); see Figure 1a. This feature is confirmed from experimental adsorption studies at (nano)crystalline HA particles^14−19^ along with computational modeling,^20−22^ encompassing very recent findings on the association of Pser molecules and ACP present in Pser-bearing CaP cements (CPCs), which is believed to underpin their bone-adhesive properties.^23,24^

Besides the overall importance given to Pser-bearing biomolecules for regulating bone-mineral growth, a prominent role is also attributed to the organic phosphate groups as CaP nucleation sites. Nearly two decades ago, Wu et al.^25^ demonstrated that ^31^P magic-angle-spinning (MAS) nuclear magnetic resonanace (NMR) spectra obtained from 8–12 days old chick embryos revealed signatures of NCP-associated PO_4_ groups devoid of contacts with Ca^2+^ in the youngest embryos, whereas those aged for ≥10 days manifested Ca^2+^···PO_4_^2–^ motifs, similar to those encountered in the Ca salt of phosphoserine, Ca[*O*-phospho-l-serine]·H_2_O (CaPser; Figure 1b).^25,26^ As highlighted in refs (25) and (27) and discussed further herein, the local ^31^P environments in the Pser and CaPser structures manifest opposite signs of their chemical-shift anisotropies (CSAs), thereby potentially rendering ^31^P MAS NMR a straightforward diagnostic tool for discriminating among organic PO_4_ groups that (do not) coordinate Ca^2+^ cations.^25^ However, follow-up studies on the local phosphate environments in embryonic bone mineral are extremely sparse, apparently being limited to density functional theory (DFT) calculations of ^31^P chemical shielding tensor parameters of various Pser···Ca^2+^ clusters.^28^ Also, the until very recent^24^ sole report^25^ on the isotropic ^31^P chemical shift of CaPser is incorrect, as elaborated on further herein. Furthermore, Kesseli et al.^29^ attributed the presence of CaPser-alike Ca^2+^···PO_4_^2–^ motifs in Pser-doped CPCs^23,24,30−33^ to their bone-adhesive properties (yet, see the critical remarks by Mathew et al.^24^).

Considering the instrumental importance of both Pser and CaPser for biomineralization problems and the mode of action of bone-bonding biomaterials, it is remarkable that the crystal structure of either compound remains poorly defined regarding its H positions because all proposed structures to date were obtained by X-ray diffraction (XRD),^26,34^ notwithstanding that neighboring Pser molecules are known to form an intermolecular H-bond network between (in particular) their carboxy and phosphate groups.^34,35^ To advance both the Pser/CaPser structure descriptions and their bearings on the ^1^H/^13^C/^31^P chemical-shift parameters, we present an array of 2D MAS NMR experimental results conducted at a fast MAS of 66 kHz for improved ^1^H resonance separation, encompassing dipolar-interaction-mediated double-quantum–single-quantum (2Q–1Q) correlation^36−40^^1^H NMR along with ^13^C{^1^H} and ^31^P{^1^H} heteronuclear correlation (HETCOR)^41^ NMR experiments. They are sensitive probes of the various ^1^H–^1^H, ^31^P–^1^H, and ^13^C–^1^H distances, which were exploited in a novel NMR crystallography protocol (outlined and discussed in Sections 3.4 and 4.1), used to assess refined structures of Pser and CaPser obtained from plane-wave DFT calculations. The internuclear-distance extraction only involves the recording of *one* 2D NMR spectrum and a *minimum* of computational efforts for each ^1^H–^1^H, ^31^P–^1^H, and ^13^C–^1^H internuclear-distance analysis.^42^

The structure refinements were evaluated further by ^1^H and ^13^C chemical shifts computed by the gauge including projector augmented wave (GIPAW) approach.^43−48^ Our NMR results are contrasted with the comparatively sparse previous solid-state NMR reports on Pser that mainly focused on the (an)isotropic ^31^P and ^13^C chemical-shift parameters,^25,27,35,49−51^ whereas most of the NMR parameters for CaPser are presented herein for the first time. We also discuss the ^31^P NMR signatures of the phosphate groups of Pser and CaPser in relation to their proposed capabilities of monitoring the very initial bone-mineral formation events.^25^

## 2 Materials and Methods

### 2.1 Preparation of CaPser

All chemicals were purchased from Sigma-Aldrich unless otherwise indicated. *O*-Phospho-l-serine (>95%, Flamma SpA) was used as received. The polycrystalline CaPser powder,^26^ identical to that utilized in our previous study,^24^ was prepared along the procedures described in refs (16) and (26) 100 mL of an aqueous solution of Pser (0.100 mol/L) was adjusted to pH = 4.3 by KOH(aq). 10 mL of 1.00 mol/L CaSO_4_(aq) was then added slowly under constant stirring, whereupon CaPser was precipitated by slowly increasing the pH value of the solution to 7.4 by dropwise addition of KOH(aq). The thereby formed white powder of CaPser was allowed to mature by keeping the solution and precipitate under constant stirring for 5 days. The powder was separated by centrifugation, washed three times with distilled water, and dried at 60 °C for 24 h. Powder XRD confirmed the phase purity of the CaPser specimen (data not shown).

### 2.2 Solid-State NMR Experimentation

All solid-state NMR experimentations utilized a Bruker Avance-III spectrometer and a magnetic field (*B*_0_) of 14.1 T (giving ^1^H, ^13^C, and ^31^P Larmor frequencies of −600.1, −150.9, and −242.9 MHz, respectively). 1.3 mm ZrO_2_ rotors were filled with Pser or CaPser powders and were spun at the MAS rate ν_r_ = 66.00 kHz. These conditions apply throughout, except for some routine ^13^C/^31^P NMR experiments (Section 2.2.1).

Resonance offsets were minimized by positioning each radio-frequency (rf) carrier ^1^H/^13^C/^31^P frequency at the mid of the NMR-signal region throughout. For achieving absorptive 2D NMR peaks with frequency-sign discrimination along the indirect dimension, all 2D NMR acquisitions implemented the States-TPPI procedure,^52^ where each number of *t*_1_ increments stated below refers to that of *each* real/imaginary data set of the hypercomplex protocol. The lowest contour levels employed for the 2D NMR spectra presented herein range between 2% and 5% of the maximum NMR intensity, except for the ^13^C{^1^H} HETCOR spectra (10%). ^1^H/^13^C and ^31^P chemical shifts are quoted relative to neat tetramethylsilane (TMS) and 85% H_3_PO_4_(aq), respectively.

#### 2.2.1 1D MAS NMR Experiments

Single-pulse ^1^H spectra were recorded using 90° rf pulses operating at the ^1^H nutation frequency ν_H_ ≈ 104 kHz, 16 (Pser) and 64 (CaPser) accumulated NMR-signal transients, and relaxation delays (τ_relax_) of 2.0 s. Single-pulse ^31^P NMR spectra from Pser and CaPser were recorded at *B*_0_ = 9.4 T (−162.0 MHz ^31^P Larmor frequency) and ν_r_ = 14.00 kHz (4 mm rotors) using 90° rf pulses operating at the ^31^P nutation frequency ν_P_ = 85 kHz, τ_relax_ = 20 min, and 512 accumulated transients. SPINAL-64 proton decoupling^53^ at ν_H_ = 84 kHz was applied during the ^31^P NMR signal detection. ^1^H → ^13^C cross-polarization (CP) MAS NMR spectra were recorded at *B*_0_ = 14.1 T and ν_r_ = 14.00 kHz using a contact period (τ_CP_) of 1.285 ms at the zero-quantum Hartmann–Hahn condition,^54^ ν_H_ = ν_C_ + ν_*r*_, where ν_C_ was ramped linearly^55^ by ±3.7 kHz around ν_C_ = 37 kHz. The ^1^H 90° rf-pulse length was 2.9 μs, and 512 transients with τ_relax_ = 4.0 s were recorded using SPINAL-64 decoupling at ν_H_ = 90 kHz during the ^13^C NMR-signal detection.

^1^H → ^31^P CP (Pser) and single-pulse (CaPser) MAS NMR spectra were also recorded at a slow MAS of ν_r_ = 2.00 kHz and ν_r_ = 3.50 kHz, respectively, using otherwise similar experimental conditions as those described above. These NMR spectra, presented in Figure S3, were used for deriving the ^31^P CSA parameters by using well-established iterative fitting procedures.^56^

#### 2.2.2 2D MAS NMR Experiments

All 2D HETCOR NMR acquisitions utilized ^1^H → ^13^C or ^1^H → ^31^P CP at the double-quantum Hartmann–Hahn condition,^54^ ν_H_ + ν_C_ = ν_r_ and ν_H_ + ν_P_ = ν_r_, respectively, which involved ramped CP of ν_C_ = 50 ± 5 kHz for ^13^C (ν_H_ = 16 kHz) and ν_P_ = 40 ± 4 kHz for ^31^P (ν_H_ = 26 kHz), a 2.4 μs 90°^1^H pulse, and continuous-wave (CW) ^1^H decoupling at the rotary resonance condition^57^ ν_H_ = ν_r_/2 = 33 kHz. The ^13^C{^1^H} HETCOR NMR spectra were recorded with τ_CP_ = 100 μs, τ_relax_ = 1.5 s, and dwell times of Δ*t*_2_ = τ_r_ = 15.15 μs and Δ*t*_1_ = 6τ_r_ (Pser) or Δ*t*_1_ = 8τ_r_ (CaPser), where τ_r_ = ν_r_^–1^ is the rotor period. 35(*t*_1_) × 2628(*t*_2_) (Pser) and 30 × 1970 (CaPser) time points were collected with 512 (Pser) and 1024 (CaPser) accumulated transients per *t*_1_ value. The 2D NMR data sets were zero-filled to 128 *t*_1_ points, along with 8192 (Pser) and 4096 (CaPser) *t*_2_ points, and were apodized by a cos^2^ and an exponential function along the indirect and direct dimensions, respectively, with the latter giving a 50 and 60 Hz full width at half-maximum (fwhm) Lorentzian broadening for Pser and CaPser, respectively.

^31^P{^1^H} HETCOR NMR data sets comprising 64 × 1643 (Pser) and 24 × 1643 (CaPser) *t*_1_ × *t*_2_ points were recorded with τ_relax_ = 1.5 s using Δ*t*_2_ = 2τ_r_ along with Δ*t*_1_ = 6τ_r_ (Pser) and Δ*t*_1_ = 8τ_r_ (CaPser). For Pser/CaPser, 128/1024 and 8/32 signal transients were accumulated per *t*_1_-value for the NMR acquisitions with τ_CP_ = τ_r_ = 15.15 μs and τ_CP_ = 1.000 ms, respectively. The 2D grids were zero-filled to 8192 *t*_2_ points and 512 (Pser) or 128 (CaPser) *t*_1_ points, followed by apodization by cos^2^ and exponential functions along the indirect and direct dimensions, respectively (giving fwhm broadenings of 10 Hz for Pser and 20 Hz for CaPser).

2Q–1Q ^1^H NMR correlation spectra were recorded with the 2D NMR protocol shown in Figure 1a of ref (58). 2Q coherence (2QC) excitation/reconversion was accomplished by the shortest BaBa dipolar recoupling scheme that extends over one sole rotor period,^58,59^ thereby giving 2Q excitation (τ_exc_) and reconversion (τ_rec_) intervals of τ_exc_ = τ_exc_ = τ_r_ = 15.15 μs. The ^1^H nutation frequency was ν_H_ ≈ 210 kHz for the 90^°^ dipolar recoupling pulses of a duration of 1.20 μs. The 2D NMR acquisitions employed τ_relax_ = 2.25 s, along with the following parameters: for Pser, 36(*t*_1_) × 3000(*t*_2_) time points were acquired with dwell times of {Δ*t*_1_ = 2τ_*r*_; Δ*t*_2_ = 3.6 μs} and 768 accumulated transients/*t*_1_-value; for CaPser, 75 × 850 time points were acquired with dwell times of {Δ*t*_1_ = 3τ_r_; Δ*t*_2_ = τ_r_} and 128 accumulated transients/*t*_1_-value. The 2D data sets were zero-filled to 256 × 16,384 (Pser) and 256 × 4096 (CaPser) points and apodized by a cos^2^ function along the indirect dimension; no apodization was applied along the direct dimension for the NMR experiment on Pser, while an exponential 5 Hz fwhm Lorentzian broadening was applied for CaPser.

While the rather crude BaBa scheme employed herein offers no chemical-shift compensation during the ^1^H–^1^H dipolar recoupling and superior BaBa incarnations exist,^58,60^ they cannot be utilized for quantitative distance analyses because they demand too long (minimum) 2QC excitation periods (Section 3.4.1) that lead to non-quantitative 2QC intensities from the strongest ^1^H–^1^H interactions in the present molecules. Numerical simulations (not shown) suggested the absence of chemical-shift compensation to be unproblematic for our BaBa implementation, as was further corroborated by complementary 2Q–1Q correlation NMR experiments performed on Pser and CaPser with the chemical-shift compensated [*S*R2_2_^1^] scheme^61,62^ for 2QC excitation and reconversion (τ_exc_ = τ_rec_ = 60.6 μs); while truly quantitative analyses were precluded for these experiments, the integrated 2Q–1Q NMR intensities for many ^1^H–^1^H pairs agreed well with those from the BaBa-derived results (see Section 3.8).

### 2.3 DFT Calculations

Energy minimizations by first-principles DFT calculations were carried out with the CASTEP software^63^ (version 19.11) and the Perdew–Burke–Ernzerhof (PBE) functional^64^ with on-the-fly-generated ultrasoft pseudopotentials^65^ and a plane-wave basis set.^66^ The Tkatchenko and Scheffler method was employed for dispersion corrections.^67^ The proton positions were adjusted during the energy minimizations of the Pser (CCDC identifier: SERPOP01) and CaPser crystal structures, which conform to the respective space groups *P*2_1_2_1_2_1_ (ref (34)) and *P*2_1_ (ref (26)). All other atom positions remained fixed, as well as the unit-cell axis lengths/directions (calculations with variable cell lengths are sometimes employed^68,69^). The {*a*, *b*, *c*} unit-cell lengths are {7.737, 10.167, 9.136} Å and {5.534, 12.759, 5.740} Å for Pser and CaPser, respectively,^26,34^ while the {α, β, γ} angles for CaPser are {90°, 104.77°, 90°}.

The ^1^H, ^13^C, and ^31^P chemical shielding values were calculated for both XRD structures of Pser and CaPser before and after the optimizations by using the GIPAW method.^43,44^ For both the DFT energy optimizations and the GIPAW shielding-parameter calculations, plane-wave energy cutoffs of 1100 eV and 1200 eV were employed for Pser and CaPser, respectively, along with a Monkhorst–Pack *k*-point grid^70^ with a maximum spacing of 0.05 Å^–1^ in the reciprocal space.

For the unique ^31^P site and each ^1^H^*j*^ and ^13^C^*j*^ site in the Pser/CaPser structure, the respective DFT/GIPAW-derived principal values {σ_*xx*_^P^, σ_*yy*_^P^, σ_*zz*_^P^}, {σ_*xx*_^H*j*^, σ_*yy*_^H*j*^, σ_*zz*_^H*j*^}, and {σ_*xx*_^C*j*^, σ_*yy*_^C*j*^, σ_*zz*_^C*j*^} of the second-rank chemical shielding tensors were converted into the corresponding sets of chemical-shift values, {δ_*xx*_^S*j*^, δ_*yy*_^S*j*^, δ_*zz*_^S*j*^}, by using the expression^46−48^
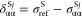
1for each component αα = {*xx*, *yy*, *zz*} and S = {^1^H, ^13^C, ^31^P}. Herein, we employ a chemical-shift scale throughout, where low (high) chemical shifts correspond to shielded (deshielded) nuclei.^71−73^ The principal-value triplet {δ_*xx*_^S*j*^, δ_*yy*_^S*j*^, δ_*zz*_^S*j*^} of each ^1^H^*j*^, ^13^C^*j*^, and ^31^P shift tensor obeys^71,73−75^
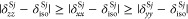
2and relates to the respective *isotropic* (δ_iso_^S*j*^) and *anisotropic* (δ_aniso_^S*j*^) *chemical shifts* by^71,74,75^
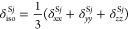
3and
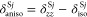
4respectively, whereas the asymmetry parameter (η^S*j*^) is defined by
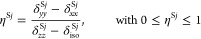
5Other definitions of δ_aniso_ and η are encountered in the literature,^72,73^ with the most common one^73^ yielding a higher anisotropy value (by 3/2) relative to that of eq 4. All our comparisons with previously published CSA parameters are made after their conversion into eqs 4 and 5.

For ^1^H and ^13^C, each shielding-to-shift conversion term in eq 1, σ_ref_^H^ = 30.445 ppm and σ_ref_^C^ = 172.346 ppm, was obtained by a linear regression that minimized the difference between the sets of calculated and experimental isotropic ^1^H and ^13^C chemical shifts, when utilizing all data available from *both* Pser and CaPser. The correlation coefficients were *R*^2^ = 0.996 and *R*^2^ = 0.999 for ^1^H and ^13^C, respectively. However, this approach was precluded for deducing the value of σ_ref_^P^, for which we pragmatically equated the DFT-derived isotropic ^31^P chemical shift with that obtained by NMR for Pser (δ_iso_^P^ = 0.0 ppm; σ_ref_^P^ = 294.85 ppm); this action had no bearings on the accuracy/precision of the {δ_aniso_^P^; η^P^} data, which remain independent of the precise σ_ref_^P^ value.

## 3 Results

### 3.1 ^1^H, ^13^C, and ^31^P MAS NMR

Here, we discuss the ^13^C, ^1^H, and ^31^P MAS NMR results compiled in Table 1. While some heavily overlapping ^1^H NMR peaks could not be assigned unambiguously on the basis of these NMR spectra alone, they were deduced by the 2D NMR experiments and DFT/GIPAW calculations discussed in the following sections.

**Table 1:** ^1^H, ^13^C, and ^31^P Chemical Shifts Obtained by NMR and DFT/GIPAW Calculations^a^

	Pser	CaPser
site	δ_iso_ (ppm)	δ_iso_ (ppm)
^13^**C**		
**C**OOH	170.7(169.5)	174.0(171.6)
**C**H_2_	64.2(66.3)	64.1(66.1)
**C**H	55.3(54.9)	56.9(56.6)
^1^**H**^b^		
COO**H**	16.7(16.9)	
PO**H**	12.5(12.5)	
N**H**_3_	8.2(8.2)	8.2(8.1)
C**H**^R^H	5.1(4.0)	4.7(4.8)
CH**H**^S^	4.2(4.2)	3.7(3.9)
C**H**	3.9(4.0)	5.2(5.1)
**H**_2_O		5.2(4.6)
^31^**P**		
**P**	0.0(0.0)^c^	–1.0(0.1)^c^

a ^13^C, ^1^H, and ^31^P isotropic chemical shifts (δ_iso_) obtained by either NMR or DFT/GIPAW calculations (values within parentheses) for the nuclear site typeset in boldface. The uncertainties (±1σ) of the NMR-derived shifts are ±0.1 ppm (^13^C and ^31^P) and ±0.15 ppm (^1^H).

b The two crystallographically inequivalent methylene proton sites are denoted by superscripts “R” and “S”.^126^ Note that the NH_3_^+^ and H_2_O moieties feature rapid molecular motions, such that only the average chemical shifts of the crystallographically inequivalent ^1^H sites are observed by NMR. Hence, the listed DFT/GIPAW-generated ^1^H chemical shifts are average values over the following shifts: {2.8, 6.8} ppm for **H**_2_O, and {6.3, 8.9, 9.3} ppm (Pser) and {5.2, 7.4, 11.6} ppm (CaPser) for N**H**_3_^+^.

c Only the *difference* between the DFT/GIPAW-derived chemical shifts of Pser and CaPser may be compared because δ_iso_^P^ of the ^31^P site of Pser was equated to the experimental result (Section 2.3). The calculated chemical shifts are only defined within an unknown constant (δ_iso_^P^ + *C*).

**Figure 2:**
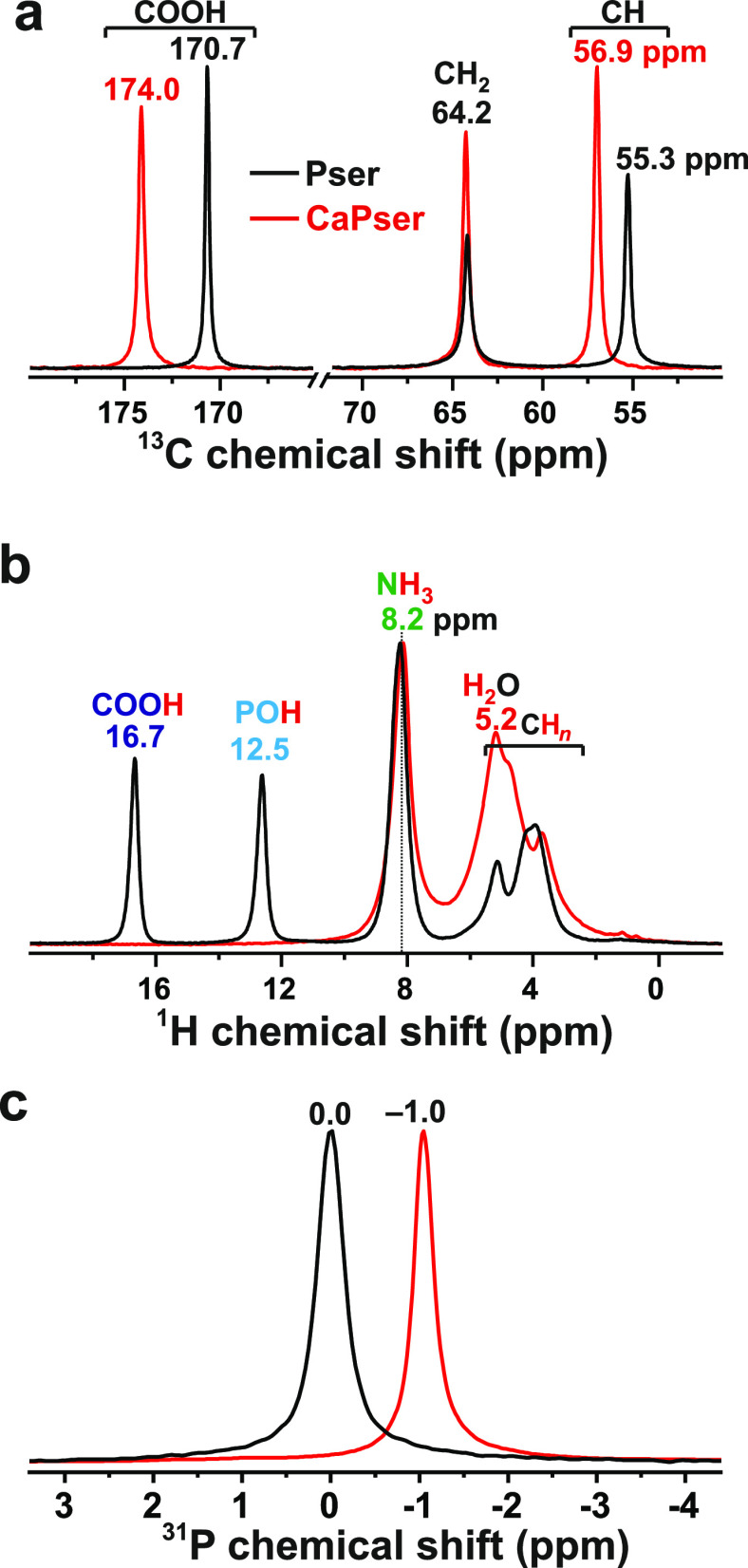
Experimental MAS NMR spectra recorded from polycrystalline powders of Pser (black traces) and CaPser (red traces) from the following nuclei, with the experimental {*B*_0_; ν_r_} conditions given in parentheses: (a) ^13^C (14.1 T; 14.00 kHz); (b) ^1^H (14.1 T; 66.00 kHz); (c) ^31^P (9.4 T; 14.00 kHz). Assignments and maxima of the NMR peaks are provided at the top of each NMR spectrum. The NMR results in (a) were obtained using ^1^H → ^13^C CP, and those in (b,c) were excited directly by single rf pulses.

Figure 2a presents the ^13^C MAS NMR spectra recorded by ^1^H → ^13^C CP from the Pser and CaPser powders. While the two molecules share the same ^13^C isotropic chemical shift (δ_C_ ≈ 64.2 ppm) of their **C**H_2_ (C^β^) moieties, CaPser reveals a slightly higher shift (δ_C_ = 56.9 ppm) of the **C**H (C^α^) site relative to its Pser counterpart (δ_C_ = 55.3 ppm). Here and onward, the boldface typesetting indicates the particular structural site considered of the as-specified functional group. Owing mainly to their distinct protonation states—COOH for Pser and COO^–^ for CaPser—but also stemming from their distinct intermolecular H-bond constellations, the largest chemical-shift difference (4.5 ppm) is observed among the carboxy groups, whose ^13^C sites resonate at 174.0 ppm and 170.7 ppm for CaPser and Pser, respectively. The latter shift accords very well with that of δ_C_ = 171.0 ppm reported by Tekely and co-workers.^35^ However, we are not aware of any previously reported ^13^C chemical shifts for the aliphatic carbon sites of Pser or *any* δ_C_ data for the ^13^C sites of polycrystalline CaPser, except for our previous work^24^ (yet, see the related report of ref (27)).

The {^13^CO, ^13^C^α^, ^13^C^β^} chemical shifts of Pser/CaPser of Table 1 may be contrasted with those of l-serine (Figure S1), whose shifts δ_C_ = {175.2, 55.8, 63.0} ppm are in excellent agreement with those of δ_C_ = {175.1, 55.6, 62.9} ppm reported previously by Ye et al.^76^ The zwitterionic form of l-serine renders the carboxy site unprotonated, thereby manifesting a chemical shift closer to that of CaPser than to its ^13^**C**OOH counterpart of Pser, along the expected chemical-shift trend upon deprotonation of carboxy moieties.^27,35,77−79^ In contrast, the ^13^C^α^ and ^13^C^β^ sites reveal very similar chemical shifts, where the replacement of the OH group of l-serine with the phosphate ester results only in a (very) modest chemical-shift alteration of +1.3 ppm for the ^13^C^β^ site.

We next turn to the ^1^H NMR responses observed from Pser and CaPser in Figure 2b. With the independent experimental 2D NMR and computational DFT/GIPAW results, the well-resolved NMR peaks at 16.7 and 12.5 ppm are readily assigned to the protons of the COOH and PO_3_(OH) moieties, respectively, whereas both Pser and CaPser share N**H**_3_ resonances at 8.2 ppm (Figure 2b). All these chemical-shift values accord well with those reported for Pser by Potrzebowski et al.^35^ at 9.4 T and 32 kHz MAS. However, although the ^1^H NMR spectrum of Figure 2b, which was recorded at *B*_0_ = 14.1 T and a faster MAS rate of 66.00 kHz, offers a markedly enhanced spectral resolution in the aliphatic region compared to that of ref (35), broadening from homonuclear ^1^H–^1^H interactions precludes unambiguous chemical-shift assignments of the altogether three NMR peaks of the C**H** and C**H**_2_ groups. For CaPser, these NMR signals additionally overlap with that of the structure-bound ^1^**H**_2_O molecule (Figure 2b).

Figure 2c displays the ^31^P MAS NMR spectra from the Pser and CaPser samples, both of which reveal one narrow NMR signal at the isotropic ^31^P chemical shifts (δ_P_) of 0.0 ppm and −1.0 ppm, respectively.^24^ The latter may be compared with the distinctly different value δ_P_ = 1.3 ppm from Wu et al.,^25^ which to our knowledge remained as the sole ^31^P shift reported from CaPser up to very recently.^24^ However, the herein observed result δ_P_ = 0.0 ppm for Pser may be contrasted with several previous reports on δ_P_: 0.2,^25^ 0.3,^50^ 0.6,^27^ and −0.9 ppm,^35^ as well as the shifts of 0.67 ppm (MAS NMR) and 0.33 ppm (single crystal) obtained by Kohler and Klein.^49^ Altogether, this corresponds to a chemical-shift scatter exceeding >1.5 ppm among various literature sources. We claim the herein reported shifts to be more accurate than previous estimates because (i) we have observed a high reproducibility of the ^31^P isotropic chemical shifts observed from both Pser and CaPser, whose occurrence as minor components in CPCs prepared from α-Ca_3_(PO_4_)_2_ and Pser^24^ accords within ±0.1 ppm among numerous specimens; (ii) the CPCs also comprise additional inorganic crystalline CaP phases with well-defined chemical shifts^24^ that constitute internal standards for validating the accuracy and precision of the shifts δ_P_ = 0.0 ± 0.1 ppm and δ_P_ = −1.0 ± 0.1 ppm observed by us for Pser and CaPser, respectively.

### 3.2 DFT/GIPAW-Derived Isotropic Chemical Shifts

**Figure 3:**
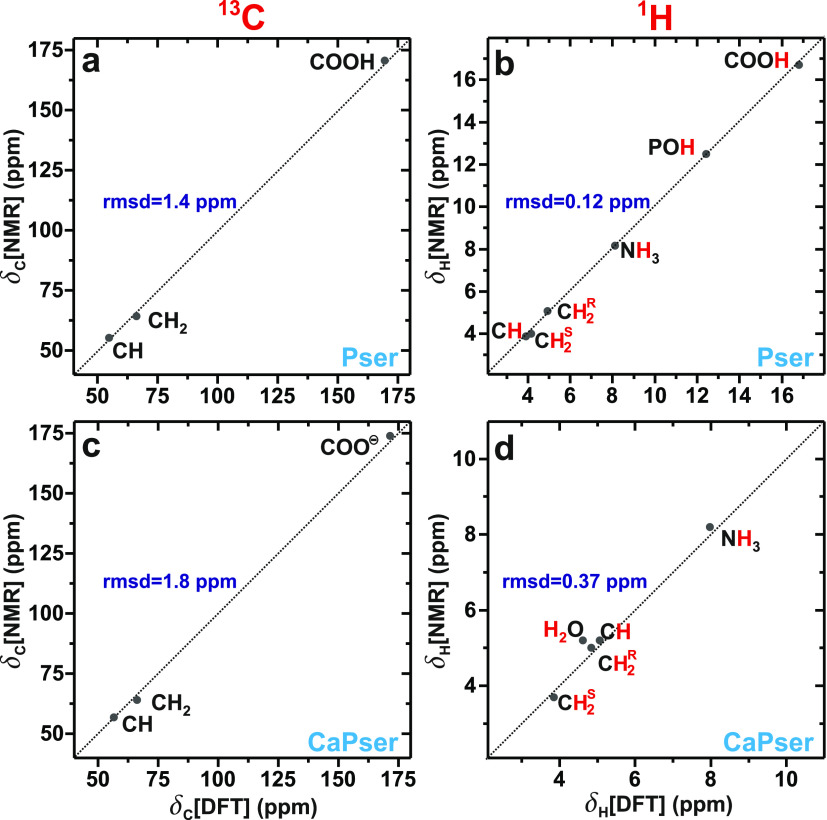
(a,c) ^13^C and (b,d) ^1^H chemical-shift correlations, where the shifts from NMR (vertical axis) and DFT/GIPAW (horizontal axis) are plotted for (a,b) Pser and (c,d) CaPser. Each dotted diagonal line represents the result of a perfect correlation, the deviation from which the as-indicated rmsd value was calculated.

Here, we contrast the experimentally determined δ_H_ and δ_C_ values with those predicted by DFT/GIPAW calculations applied to each energy-optimized Pser and CaPser structure (Table 1). Figure 3 evidences an excellent agreement between the NMR and DFT/GIPAW data sets, which do moreover not reveal any obvious systematic deviations. Root-mean-square deviations (rmsds) of 1.4 ppm and 1.8 ppm are observed for the ^13^C chemical shifts of Pser and CaPser, respectively, whereas the corresponding rmsd values for the ^1^H shifts are 0.12 ppm and 0.37 ppm for Pser and CaPser; these data translate into the typical relative errors of ≈2% and 3–4% for the ^13^C and ^1^H shift predictions, respectively.

The accuracy of our ^1^H/^13^C shift predictions compares favorably with current state-of-the art DFT calculations.^45,47,48,80^ The comparatively higher *relative* errors of the DFT/GIPAW-derived ^1^H chemical shifts from the experimental data of Table 1 stem partially from the larger uncertainties of the experimental values, which are less accurate than those for ^13^C by the combination of much broader ^1^H resonances and smaller magnitudes of the ^1^H chemical shifts. Nonetheless, these deviations are well bracketed by the experimental and computational uncertainties, except for the ^1^**H**_2_O shift of CaPser, whose rather large shift difference of 0.6 ppm corresponds to a relative discrepancy of 11%. The corresponding GIPAW-generated chemical shifts of the XRD structures of Pser and CaPser are listed in Table S1: they yield markedly worse agreements with the NMR experiments than those observed after the structure refinements.

Notably, both the NMR and DFT/GIPAW-derived ^1^H chemical-shift sets listed in Table 1 reveal that the two crystallographically inequivalent protons of the methylene moiety differ by ≈1 ppm for each Pser and CaPser structure, whereas both manifest very similar chemical shifts at ≈5.0 ppm and ≈4.0 ppm for the C**H**^R^H and CH**H**^S^ sites, respectively. The main distinction among the Pser and CaPser molecules concerns their C**H** environments, whose chemical shifts are close to those of C**H**^R^H and CH**H**^S^ for CaPser and Pser, respectively, yet being slightly higher and lower than the corresponding methylene shifts (Table 1). Hence, in the NMR spectrum from CaPser (Pser), the C**H** resonance appears to the left (right) of its methylene counterpart; see Table 1 and the 2D NMR spectra discussed in the following sections. These trends were born out by both the experimental and modeled chemical shifts of Table 1. We expect our NMR-signal assignments of the ^1^H sites of Pser and CaPser to be helpful for future NMR studies, not the least in consideration that a very recent report on the ^1^H NMR peak widths observed at very fast MAS from polycrystalline Pser employed incorrect NMR peak assignments of the aliphatic C**H** and C**H**_2_ sites.^81^

### 3.3 ^13^C–^1^H and ^31^P–^1^H Proximities by HETCOR NMR

Here, we discuss qualitatively the ^13^C{^1^H} and ^31^P{^1^H} HETCOR NMR results, which reveal each pairwise ^1^H–^13^C and ^1^H–^31^P proximity in the Pser/CaPser structure by a correlation peak appearing at the 2D NMR spectral coordinate {δ_H_, δ_C_} and {δ_H_, δ_P_}, respectively.^36,41^ In these 2D NMR spectra, the ^1^H and ^13^C/^31^P chemical shifts are encoded along the vertical (indirect) and horizontal (direct) spectral dimensions, respectively.

As discussed further in Sections 3.4–3.6, the S{^1^H} HETCOR correlation signal intensity relates to the *heteronuclear* through-space *dipolar-coupling constant* (in units of Hz), which for two specific sites (atom coordinates) *m* and *n* of a S_*m*_^*j*^–^1^H_*n*_^*k*^ spin pair is given by
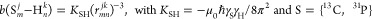
6where *r*_*mn*_^*jk*^ is the internuclear S_*m*_^*j*^–^1^H_*n*_^*k*^ distance, while γ_H_ and γ_S_ denote the magnetogyric ratios of ^1^H and S, respectively.^36,40,71^ Henceforth, each index *j* and *k* denotes a “magnetically unique/equivalent” S and ^1^H site in the molecule, respectively, which encompasses crystallographically inequivalent sites that by rapid molecular dynamics are rendered equivalent from the NMR viewpoint. Considering ^13^C, *j* may represent either of {**C**OOH, **C**H, **C**H_2_}. Similarly, ^1^H^*k*^ is the selected species *k* out of {PO**H**, COO**H**, N**H**_3_, C**H**, C**H**^R^H, CH**H**^S^} and {N**H**_3_, C**H**, C**H**^R^H, CH**H**^S^, **H**_2_O} in Pser and CaPser, respectively. Since there is only one unique P site in each Pser and CaPser crystal structure, we onward drop the superscript when considering ^31^P–^1^H^*k*^ interactions.

**Figure 4:**
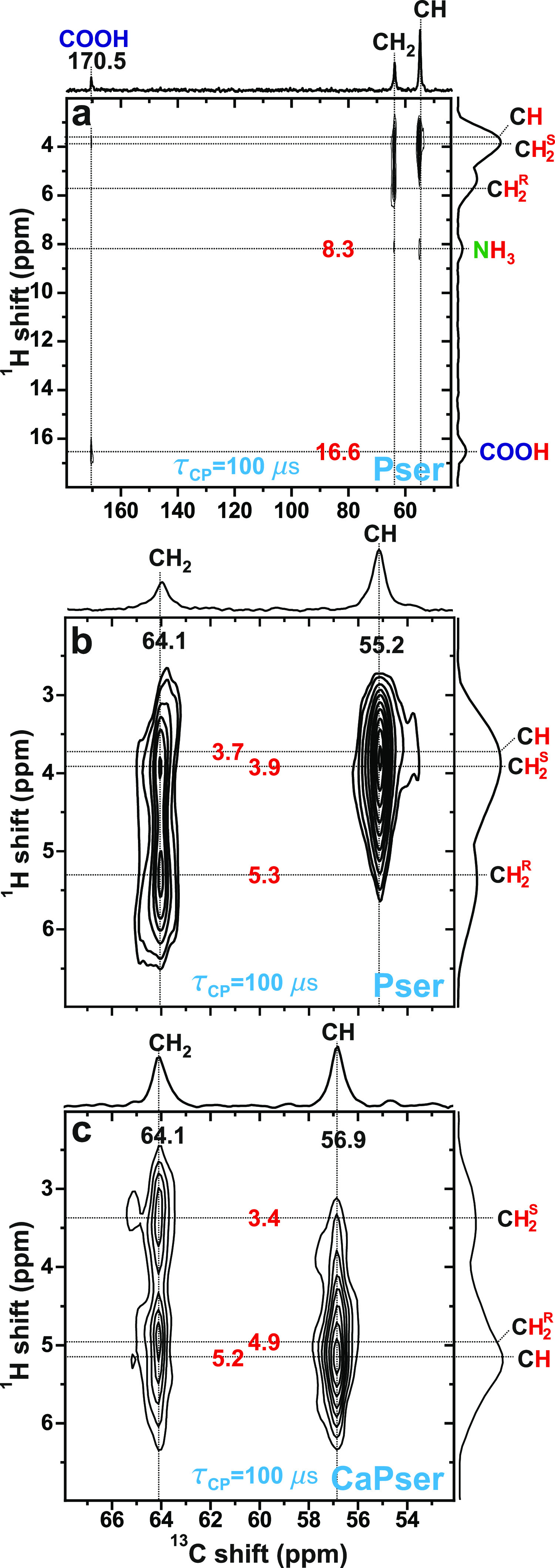
^13^C{^1^H} HETCOR NMR spectra recorded from (a,b) Pser and (c) CaPser at *B*_0_ = 14.1 T and ν_r_ = 66.00 kHz MAS, obtained for a ^1^H → ^13^C CP contact time period of 100 μs. The spectrum in (b) shows a zoom around the aliphatic spectral region of that displayed in (a). Each 2D NMR spectrum is shown together with ^1^H NMR peak assignments and the projections along the ^13^C (horizontal; top) and ^1^H (vertical; right) spectral dimensions.

Figure 4 shows ^1^H → ^13^C CP-based ^13^C{^1^H} HETCOR NMR spectra recorded from (a,b) Pser and (c) CaPser. From the relatively short contact period of τ_CP_ = 100 μs employed, the most prominent 2D NMR-peak intensities are expected from directly bonded ^13^C and ^1^H sites, that is, the aliphatic ^13^C^α^ and ^13^C^β^ sites resonating at ≈56 ppm and at 64 ppm, respectively. Each CH and CH_2_ moiety is unambiguously identified on the basis of its strong ^13^C correlation with ^1^H resonances from one and two inequivalent protons, respectively. The ^13^C{^1^H} HETCOR NMR spectra of Figure 4b,c confirm the sharing of near-equal chemical shifts of each C**H**^R^H and CH**H**^S^ moiety among the Pser and CaPser structures, while their methylene protons exhibit different chemical shifts within each molecule: both Pser and CaPser reveal slightly lower shifts for the CH**H**^S^ sites (3.9/3.4 ppm) than those listed in Table 1, thereby translating into a slightly larger chemical-shift difference of ≈1.5 ppm relative to that of C**H**^R^H. Additionally, the HETCOR NMR spectrum observed from Pser (Figure 4a) reveals two very weak correlations at the {δ_H_, δ_C_} = {16.6, 170.5} ppm and {δ_H_, δ_C_} = {8.3, 55.2} ppm shift pairs, which stem from the longer-range **C**OO**H** and **C**H···N**H**_3_ contacts, respectively. We remind that all ^1^H → ^13^C magnetization transfers occur through space, as opposed to through chemical bonds, as for the J interaction.^36,71^

**Figure 5:**
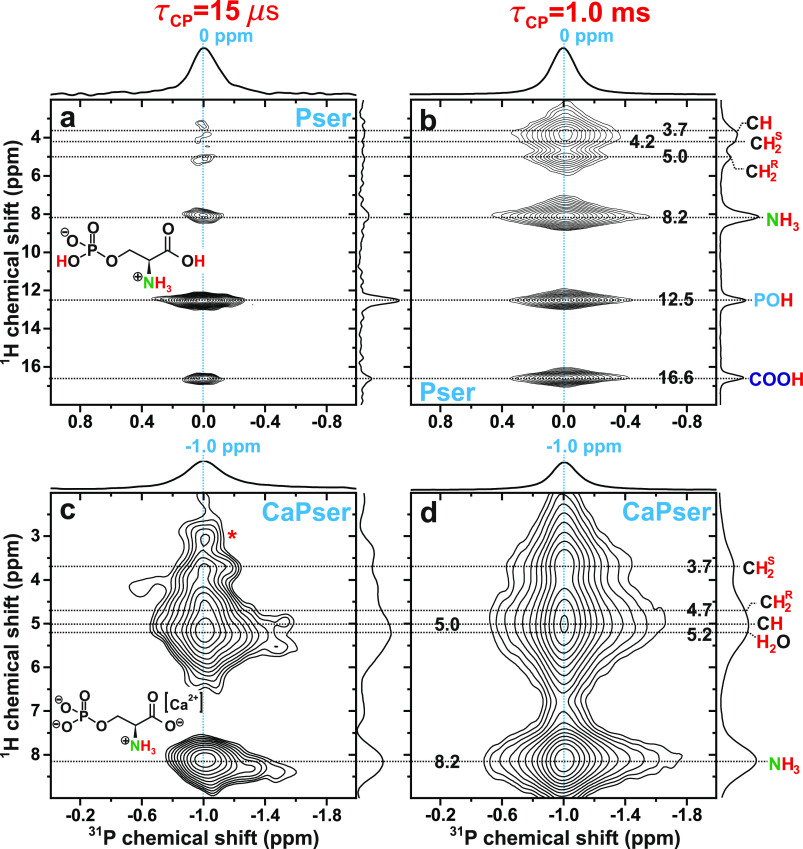
^31^P{^1^H} HETCOR NMR spectra recorded from (a,b) Pser and (c,d) CaPser at 66.00 kHz MAS with ^1^H → ^31^P CP contact time periods of (a,c) τ_CP_ = 15.15 μs and (b,d) τ_CP_ = 1.000 ms. Each 2D NMR spectrum is shown together with ^1^H NMR peak assignments and the projections along the ^1^H (vertical; right) and ^31^P (horizontal; top) spectral dimensions. The NMR peak marked by an asterisk in (c) is an artifact of unknown origin.

We next consider the ^31^P{^1^H} HETCOR NMR spectra displayed in Figure 5a,b, which were recorded from Pser by using “short” and “long” contact periods of 15 μs (left panel) and 1.000 ms (right panel), respectively. As expected from the very short period τ_CP_ = 15 μs, the correlation signal from the **P**O**H** moiety of Pser dominates the HETCOR NMR spectrum of Figure 5a. However, weak but significant 2D NMR peak intensities are visible at the ^1^H shifts of 16.6 ppm and 8.2 ppm, which originate from magnetization transfers from protons of the nearest-neighboring amino group to the ^31^**P**O_4_ site, as well as *intermolecular***P**···COO**H** contacts due to H-bonding.^27,34,35^ For the longer contact period of τ_CP_ = 1.000 ms, strong heteronuclear contacts are evident between ^31^P and all protons of the molecule, where N**H**_3_···^31^**P** manifests the overall most intense correlation due to its triplet of contributing protons. Moreover, comparable signal intensities are observed from the intramolecular **P**O**H** and intermolecular COO**H**···^31^**P** contacts.

Figure 5c,d depicts the corresponding ^31^P{^1^H} HETCOR NMR spectra obtained from CaPser. Note that because its PO_4_ group is not protonated (in contrast with that of Pser), all observed NMR correlations now involve protons from neighboring functional groups. The two most intense correlations of the 2D NMR spectrum recorded using τ_CP_ = 15 μs stem from ^31^P contacts with protons of either the amino group (δ_H_ = 8.2 ppm) or the nearby water molecule (δ_H_ = 5.2 ppm; the shortest **P**···**H**_2_O distance is 269 pm; see Table S3), but significant magnetization transfers are also discernible from the methylene protons resonating at 3.7/4.7 ppm. Besides an overall enhanced signal intensity observed in the HETCOR spectrum recorded using τ_CP_ = 1.000 ms and shown in Figure 5d, its most notable distinction to that of Figure 5c concerns a minor peak-maximum displacement of the broad NMR-signal ridge that extends across 4.5–6 ppm, which results from heavily overlapping correlation signals between ^31^P with each of C**H**, **H**_2_O, and C**H**^R^H; compare the ^1^H projections of Figure 5c,d. While the signal maximum (δ_H_ = 5.2 ppm) in Figure 5c reflects predominantly the comparatively short **P**···**H**_2_O distance, the minor displacement to δ_H_ = 5.0 ppm in Figure 5d stems from the emphasized **P**···C**H** correlation peak centered at {δ_H_, δ_P_} = {5.2, −1.0} ppm. This 2D NMR signal cannot be unambiguously identified in the 2D NMR spectrum recorded with τ_CP_ = 15 μs (Figure 5c) because it is swamped by the intense ^1^**H**_2_O resonance, whereas Figure 5d features an emphasized **P**···C**H** 2D NMR signal but a diminished **P**···**H**_2_O counterpart due to *T*_1ρ_ spin relaxation during CP.

### 3.4 General Heteronuclear Distance-Determination Procedure

Here, we outline the protocol employed for our ^31^P–^1^H and ^13^C–^1^H distance analyses, while that for ^1^H–^1^H is essentially identical within a trivial change of notation (Section 3.8). We henceforth consider an arbitrary S^*j*^–H^*k*^ spin-1/2 pair in a structure, herein targeting the various ^13^C^*j*^–^1^H^*k*^ and ^31^P–^1^H^*k*^ pairs in either the Pser or CaPser structure. The *fractional intensity* (or *fractional dipolar contact*), *f*_NMR_(S^*j*^–H^*k*^), denotes the ratio of the corresponding integrated ^31^P{^1^H} or ^13^C{^1^H} HETCOR NMR-peak intensity relative to the *entire* integrated 2D NMR spectral intensity (*I*_tot_):^42^
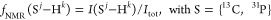
7

For a (very) short ^1^H → S CP time interval, such as that employed in Figure 5a,c, *f*_NMR_(S^*j*^–H^*k*^) is proportional to the squared *effective* coupling constant, *b*_eff_^2^(S^*j*^–H^*k*^),^37,39,42,48^ which represents the sum over the *M*_*k*_ strongest squared dipolar-interaction constants [*b*^2^(S_*m*_^*j*^–H_*n*_^*k*^)] in the structure
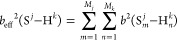
8and is directly proportional to the van Vleck dipolar second moment.^82^ The index runs over all ^1^H_*n*_^*k*^ atom coordinates of sites that are magnetically equivalent; hence, for the Pser/CaPser structures, the two methylene protons are treated with distinct indices, as opposed to those of NH_3_^+^ (Section 3.3). *b*_eff_^2^(S^*j*^–H^*k*^) relates to the squared total dipolar interaction between S^*j*^ and *all*^1^H^*k*^ structural sites from the various functional groups, *b*_eff_^2^ (tot), according to^42^
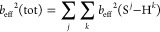
9

The *dipolar multiplicitiesM*_*j*_ and *M*_*k*_ of eq 8 are somewhat arbitrary, where we employed the *M*_*j*_/*M*_*k*_ shortest distances listed in Tables S2 and S3 in our evaluations. While the summation may be performed out to long S_*m*_^*j*^–H_*n*_^*k*^ distances (>1 nm), *b*_eff_(S^*j*^–H^*k*^) converges rapidly due to the *r*^–6^ dependence of *b*_eff_^2^(S_*m*_^*j*^–H_*n*_^*k*^) of eq 6, meaning that its value depends predominantly on the short-range S_*m*_^*j*^–H_*n*_^*k*^ interactions, which are also those mainly governing the *f*_NMR_(S^*j*^–H^*k*^) data.

The expressions eqs 8 and 9 are readily calculated from the atom coordinates of a given structure model, where we consider both the XRD-derived crystal structures for Pser/CaPser^26,34^ and their by DFT-optimized counterparts. Hence, in analogy with eq 7, each fractional dipolar contact, *f*_XRD_(S^*j*^–H^*k*^) and *f*_DFT_(S^*j*^–H^*k*^), may be derived from the set of {S_*m*_^*j*^–H_*n*_^*k*^} distances in the structure via the expression^42^
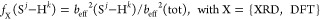
10Moreover, the NMR-derived {*f*_NMR_(S^*j*^–H^*k*^)} set may be converted into its corresponding {*r*_eff_^NMR^(S^*j*^–H^*k*^)} values by the identification *I*_tot_ = *b*_eff_^2^(tot), with *b*_eff_^2^(tot) calculated from eq 9, using the XRD-derived atom coordinates before and after refinement (or from any other known crystal structure). Hence, in direct analogy with eq 10, each NMR-derived *b*_eff_^2^(S^*j*^–H^*k*^) value is obtained by^42^
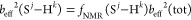
11

For a given S^*j*^–H^*k*^ pair in a structure, its effective dipolar coupling constant is related to an “effective” interatomic distance, *r*_eff_^X^(S^*j*^–H^*k*^), by^42^
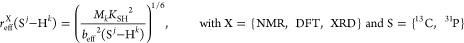
12where *K*_SH_ is defined in eq 6. By utilizing the known set of S^*j*^–H^*k*^ distances of each XRD- and DFT-derived Pser/CaPser crystal structure and combining eqs 8, 9, and 12, we calculated two sets of effective distances for S = {^13^C, ^31^P}: {*r*_eff_^DFT^(S^*j*^–H^*k*^)} and {*r*_eff_^XRD^(S^*j*^–H^*k*^)}. The validity of each such distance set may be assessed by its accordance with the NMR-derived counterpart, {*r*_eff_^NMR^(S^*j*^–H^*k*^)}. Note that the “effective” distance extracted via eq 12 was obtained from a sum over the squared dipolar coupling constants (eq 8) associated with each set of short S_*m*_^*j*^–H_*n*_^*k*^ distances encountered in the structure (Tables S2 and S3), which yields a value of *r*_eff_(S^*j*^–H^*k*^) that is intermediate of the longest and shortest distances within the set {S_*m*_^*j*^–H_*n*_^*k*^} yet closer to the shorter ones.^42^

#### 3.4.1 Prerequisites of the Protocol

Here, we discuss the prerequisites of our procedure for determining effective distances from one sole dipolar-based 2D NMR experiment, which applies to both hetero- and homonuclear effective-distance analyses (see Sections 3.7–3.8 for our ^1^H–^1^H results on Pser). A conservative prerequisite for the extraction of accurate effective internuclear S^*j*^–H^*k*^ distances via eq 7 is that
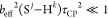
13a
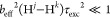
13bfor CP-based HETCOR and homonuclear 2Q–1Q correlation ^1^H NMR experiments. This demands that the detected 2D NMR signal intensity, *I*(S^*j*^–H^*k*^) (where S may be ^1^H or another spin species), remains a minor fraction of the maximum intensity [*I*_max_(S^*j*^–H^*k*^)] encountered in NMR experiments with progressively lengthened τ_CP_ or τ_exc_ intervals. For accurate internuclear-distance results, this implies in practice that
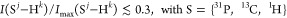
14must hold for *all* S^*j*^–H^*k*^ pairs in the system, in direct analogy with similar criteria reported from widely utilized NMR experimentation for extracting heteronuclear^83−85^ and homonuclear^37,39,60,86,87^ dipolar second moments by NMR experiments with variable recoupling intervals.

Despite that our distance-determination protocol only becomes free from systematic errors in the formal limit of eqs 13a and 13b, that is, when *I*(S^*j*^–H^*k*^)/*I*_max_(S^*j*^–H^*k*^) → 0, the criterion of eq 14 is much more forgiving. For heteronuclear applications, eqs 8 and 13a and 13b dictate the upper limit of the experimentally acceptable τ_CP_ interval, whereas for homonuclear 2QC applications, the less stringent requirement of eq 14 may be rationalized from the following properties: the buildup *rate* of 2Q coherences in multispin systems under a 2Q effective dipolar Hamiltonian is governed by the effective dipolar coupling constant of the system, whereas the maximum 2QC *amplitude* scales *approximately* as *I*_max_(H^*j*^–H^*k*^) ∼ *b*_eff_^2^(H^*j*^–H^*k*^), as deduced from analytical solutions of the 2QC generation in small systems of 2–3 spins-1/2.^37,88−90^ These rate/amplitude features of the 2QC dynamics have been confirmed both numerically and experimentally for larger spin systems^91−93^ and appear to be quite general for multiple-quantum excitation in multispin systems; for instance, they also apply to triple-quantum (3Q) excitation by either a two-spin 2Q or a three-spin 3Q dipolar Hamiltonian.^94,95^ Hence, for two spin pairs H^*j*^–H^*k*^ and H^*p*^–H^*q*^ of a system that features a relatively uniform set of dipolar interaction strengths among its various pairs, the following relationship among the 2Q–1Q NMR intensities,
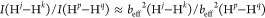
15holds reasonably well for “small” τ_exc_ values also well beyond the limit of τ_exc_ ≈ 0 (eqs 13a and 13b),^36−39,42^ thereby rationalizing eq 14. However, the precise upper limit of *I*(S^*j*^–H^*k*^)/*I*_max_(S^*j*^–H^*k*^) of eq 14 depends on the relative spin-system topology, where “dipolar truncation” effects^36−38,92^ may occur in spin systems with wide spreads of dipolar-interaction strengths.

For practical heteronuclear and homonuclear 2D NMR implementations, we recommend selecting the shortest possible dipolar recoupling period for the 2D NMR experiment, within the constraints from the signal-to-noise ratio (S/N) of the spectrum (e.g., see Section 3.6) or the sampling restrictions by the recoupling scheme (Sections 2.2 and 4.1). There are two primary reasons for this recommendation: (i) Effects from NMR relaxation, experimental rf-pulse imperfections/inhomogeneity, and dipolar truncation are minimized. (ii) The systematic error of an NMR-extracted *r*_eff_(S^*j*^–H^*k*^) value increases concurrently with the ratio {*I*(S^*j*^–H^*k*^)/*I*_max_(S^*j*^–H^*k*^)} in eq 14, which coupled with eq 9 implies that “short” (“long”) effective distances become overestimated (underestimated). Although strictly not necessary, performing another 2D NMR experiment with a (slightly) longer dipolar recoupling period may confirm the validity of the first set of estimated internuclear distances and help ensuring that both NMR experiments obeyed eq 14.

#### 3.4.2 Data Uncertainties

For highly accurate crystal structures, such as XRD data for most atom types in well-ordered structures (but disregarding the dominant *systematic* errors from the uncertain proton coordinates that are typically the subject of refinements), the relative uncertainties in the fractional dipolar contacts (eq 7) and the effective distances (eq 12) are in practice dictated by the uncertainty in the choice of cutoff distance that defines the dipolar multiplicity in eq 8. For the XRD/DFT-derived crystal structures used herein, these (relative) uncertainties are about σ(*f*_DFT_) = σ(*f*_XRD_) = 4%, which translates into relative effective-distance uncertainties of σ(*r*_eff_^DFT^) = σ(*r*_eff_^XRD^) = 0.7%. Note that although the {*f*_NMR_} data are obtained independently from the 2D NMR spectrum, the uncertainty of *b*_eff_^2^(tot) from eq 11 couples with the experimental uncertainties in the *f*_NMR_ → *r*_eff_^NMR^ conversion, such as for cases of high signal-to-noise (S/N) and well-resolved 2D NMR peaks, that is, for many—yet, not all—2Q–1Q correlation ^1^H NMR signals discussed in Section 3.7. That additional uncertainty, however, is in general negligible relative to the experimental integration errors from 2D NMR data with low/moderate S/N and partially/heavily overlapping correlation NMR peaks—such as those of the present ^13^C{^1^H} HETCOR NMR spectra.

All *f*_NMR_ results presented herein were obtained by a direct and straightforward integration of each 2D NMR peak volume across a square/rectangular chemical-shift range, which for the 2Q–1Q ^1^H correlations involved summation of the two peak intensities of each 2QC (except for the “diagonal” signals); see Sections 3.7–3.8. We did not attempt resolving heavily overlapping peaks by a formally more accurate 2D NMR spectra deconvolution (see ref (42)), partially due to the unknown ^1^H resonance shapes along the indirect spectral dimension, which have both Lorentzian and Gaussian components.

### 3.5 ^31^P–^1^H Distances

Contrasting the results for {*f*_NMR_(P–H^*k*^)} and {*f*_DFT_(P–H^*k*^)} provides a direct experimental assessment of each by DFT-refined crystal structure of Pser and CaPser. These data are listed in Table 2 and reveal an excellent agreement between the NMR and DFT-derived data for all spin pairs. As expected, from both the set of shortest P–H distances of Table S3 and the ^31^P{^1^H} HETCOR NMR spectra of Figure 5a, the **P**O**H** pair of Pser accounts for the majority of both DFT/NMR-derived fractions (≈0.53), whereas for the non-protonated phosphate group of CaPser, the contributions from the globally shortest **P**···**H**_2_O distance dominate both *f*_NMR_ and *f*_DFT_ values. However, owing to the ambiguities in separating the 2D correlation NMR peak intensities from those involving the C**H** and **H**_2_O protons (Section 3.3), we only report their summed fractions in Table 2, which account for *f*_NMR_ = 0.49 and *f*_DFT_ = 0.47; for the latter, the **P**···C**H** and **P**···**H**_2_O contacts contribute with the respective fractions *f*_DFT_ = 0.16 and *f*_DFT_ = 0.31.

**Table 2:** Effective ^31^P–^1^H Distances^a^

sites	*f*_NMR_	*f*_DFT_	*f*_XRD_	*r*_eff_^NMR^(*r*_eff_^DFT^) (pm)	Δ*r*^DFT^ (pm)	*r*_eff_^NMR^(*r*_eff_^XRD^) (pm)	Δ*r*^XRD^ (pm)
*Pser*							
COO**H**	0.116	0.125	0.038	268(265)	3	260(313)	–53
PO**H**	0.536	0.535	0.690	233(233)	0	202(194)	8
N**H**_3_	0.135	0.136	0.084	293(293)	0	305(330)	–25
C**H**^R^H^S^	0.093	0.093	0.097	278(278)	0	270(269)	2
CH^R^**H**^S^	0.084	0.083	0.068	318(318)	0	309(320)	–11
C**H**	0.036	0.028	0.023	411(429)	–18	400(431)	–31
*CaPser*							
N**H**_3_	0.260	0.278	0.245	316(313)	3	323(327)	–3
C**H**^R^H^S^	0.163	0.164	0.165	285(284)	1	291(291)	0
CH^R^**H**^S^	0.087	0.089	0.102	355(353)	2	363(353)	10
C**H** + **H**_2_O	0.490	0.469	0.488	285(287)	–2	305(305)	0

a Fractional ^31^P–^1^H dipolar contacts *f*_NMR_, *f*_DFT_, and *f*_XRD_ obtained by ^31^P{^1^H} HETCOR NMR and the XRD-derived crystal structures of Pser and CaPser before (*f*_XRD_) and after (*f*_DFT_) energy optimization by DFT, along with the respective effective ^31^P–^1^H distances {*r*_eff_^NMR^, *r*_eff_^DFT^, *r*_eff_^XRD^} calculated from eqs 8, 10, 11, and 12. Δ*r*^X^ = *r*_eff_^NMR^ – *r*_eff_^X^ (X = {DFT, XRD}) represents the deviation between the NMR-derived effective distance and that from the corresponding DFT or XRD structure. The uncertainties (±σ) of the {*f*_NMR_, *f*_DFT_, *f*_XRD_} values are {±10%, ±4%, ±4%} and those of the corresponding effective distances are {±2%, ±0.7%, ±0.7%}.

**Figure 6:**
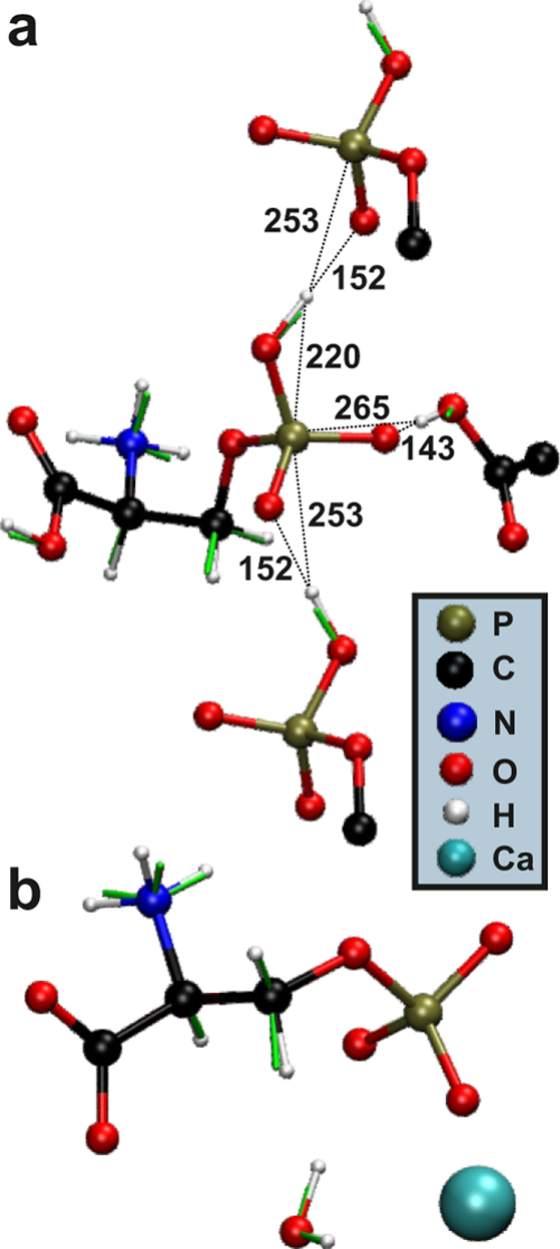
Ball-and-stick representations of the (a) Pser and (b) CaPser structures obtained by refining the H positions by DFT calculations (H atoms represented by white balls) relative to those of the XRD-derived structures,^26,34^ whose positions are represented by green bars. The numbers represent interatomic distances in pm. In (a), one carboxy and two phosphate groups of the nearest-neighboring Pser molecules are also displayed to convey the intermolecular H-bond network in the structure, where a H-bond occurs between an O site of the phosphate group and that of COO**H** in a nearest-neighboring molecule in the crystal structure (with a **P**O···COO**H** distance of 265 pm). The phosphate groups of neighboring Pser molecules manifest significantly longer intermolecular **P**···**H** distances of 253 pm relative to that of 220 pm within each **P**O_3_(O**H**) moiety.

Table 2 also lists the sets {*r*_eff_^NMR^(P–H^*k*^)} of effective distances obtained from Pser and CaPser. For Pser, the NMR-derived effective distances between ^31^P and the proton of each {PO**H**, COO**H**, N**H**_3_, C**H**^R^H, CH**H**^S^, C**H**} moiety are {233, 268, 293, 278, 318, 411} pm; they yield an essentially exact match with the {*r*_eff_^DFT^} set, with the very minor discrepancies of only a few pm remaining well within the experimental/computational uncertainties. Notably, an overall better agreement is observed between the P–H distances obtained from NMR and its DFT-derived counterpart relative to those extracted from the initial XRD structure (Table 2), as reflected in the correlation coefficients *R*^2^ = 0.989 and *R*^2^ = 0.926 between the {*r*_eff_^NMR^} set and those of {*r*_eff_^DFT^} and {*r*_eff_^XRD^}, respectively. The improvements particularly concern the *inter*molecular **P**···COO**H** contact, which constitutes the overall second shortest P–H distance and for which the resulting effective-distance pairs are {*r*_eff_^NMR^ = 268 pm; *r*_eff_^DFT^ = 265 pm} and {*r*_eff_^NMR^ = 260 pm; *r*_eff_^XRD^ = 313 pm}; see Figure 6a, which contrasts the structure before and after refinement by DFT. Note that *r*_eff_^NMR^ differs among the two sets due to the distinct values of *b*_eff_^2^(tot) used in eq 11 from the DFT and XRD structures. The results of Table 2 altogether suggest that the energy optimization by DFT significantly improved the proton positions in the Pser structure reported in ref (34), thereby in particular enhancing the description of the H-bonding between neighboring molecules (Figure 6).

An excellent agreement is also observed between the DFT-refined structure of CaPser and the ^31^P{^1^H} HETCOR NMR results (Table 2). The NMR-derived effective ^31^P–^1^H distances are {316, 285, 355, 285} pm for the corresponding {N**H**_3_^+^, C**H**^R^H, CH**H**^S^, C**H**/**H**_2_O} moieties, which all accord within 3 pm. However, the absence of protons at both the phosphate and carboxy groups restricts comparisons to the P contacts with those of the aliphatic and NH_3_^+^ protons. Overall, more modest improvements are observed from the DFT optimization of the previously reported crystal structure of CaPser^26^ (Table 2; Figure 6b), as is mirrored by their correlation coefficients *R*^2^ = 0.997 (DFT) and *R*^2^ = 0.953 (XRD) relative to the NMR results. Figure S4a,b presents correlation plots of the effective internuclear distances obtained from NMR relative to those of either the XRD- or DFT-generated structures of both the Pser and CaPser molecules. Apparently, the XRD-derived structure of CaPser^34^ is closer to the “real one” than its Pser counterpart of ref (26), as reflected by the fact that significant refinements were only observed for Pser.

### 3.6 ^13^C–^1^H Distances

From a ^13^C{^1^H} HETCOR NMR spectra analysis (Figure 4a,c) according to the procedure outlined above, a set of effective *r*_eff_^NMR^(C^*j*^–H^*k*^) distances were extracted via eq 12. The results are listed in Table S4, along with the counterparts derived from the “DFT” and “XRD” crystal structures. However, the required use of a short contact time period makes the 2D NMR spectra dominated by the ^13^C–^1^H correlation signals of the aliphatic groups. Unfortunately, our employed contact interval of τ_CP_ = 100 μs was *still too long* to obey eq 14 and enable accurate effective ^13^C–^1^H distance determinations, whereas the use of τ_CP_ ≲ 30 μs that would secure quantitative 2D NMR intensities (e.g., see refs (96) and (97)) was precluded for signal-sensitivity reasons. Consequently, the HETCOR spectra of Figure 4a,c lead to an underestimation of the effective dipolar interactions (and thereby to an overestimation of *r*_eff_^NMR^) for the methylene group of each Pser and CaPser molecule, whereas the **CH** distance—and notably that of **C**OO**H**—become underestimated. These effects are evident from the *f*_NMR_ data in Table S4: in the limit of *b*_eff_^2^τ_CP_^2^ ≪ 1 for all C^*j*^ −H^*k*^ pairs in the structure, only ^13^C NMR signals from the directly bonded **CH**, **CH**^R^H, and **C**H**H**^S^ fragments would be detected, whose respective intensities should relate *approximately* as 1:1:1 in the ^13^C{^1^H} HETCOR NMR spectrum, that is, roughly a 2:1 NMR intensity distribution among the CH_2_:CH groups.

The most notable differences among the “XRD” and “DFT” crystal structures concern their sets of effective **CH**, **CH**^R^H, and **C**H**H**^S^ distances: both XRD-derived Pser and CaPser structures reveal significantly shorter **CH** distances of ≈92 pm relative to their methylene counterparts that are 105/118 pm (Pser) and 102/107 pm (CaPser); see Table S4. Upon structural relaxation by the energy optimization, however, both the Pser and CaPser DFT models manifest **CH** distances of ≈110 pm. Somewhat surprising is that the trend of *distinct* C**H**/C**H**_2_ distances is reproduced by the ^13^C{^1^H} HETCOR NMR experiments, whose corresponding data accord overall better with those of the unrefined XRD structures in Table S4. Notably, this inference contrasts strongly with those of the P–H and H–H distance analyses (Sections 3.5 and 3.8), which consistently favored the DFT-optimized structures. We reiterate the caveat that the fractional NMR intensities {*f*_NMR_(C^*j*^–H^*k*^)} and effective distances {*r*_eff_^NMR^(C^*j*^–H^*k*^)} of Table S4 may have systematic errors due to violation of eq 14 and thereby do not offer reliable validations of either the “DFT” or “XRD” structure. Rather, the apparently shorter NMR-derived **CH** distances for both molecules most likely reflect artifacts from using too long contact intervals (*vide supra*).

### 3.7 ^1^H–^1^H Proximities Revealed by 2Q–1Q Correlation NMR

After having discussed the ^13^C–^1^H and ^31^P–^1^H proximities of the Pser and CaPser molecules deduced from heteronuclear correlation NMR, we now examine the *homonuclear*^1^H–^1^H contacts by 2Q–1Q correlation ^1^H NMR experiments.^36−40^ They rely on the creation of 2QC in pairs of nearby protons via their homonuclear ^1^H–^1^H dipolar interactions. In direct analogy with eq 6, the dipolar coupling constant of two specific sites *m* and *n* of a ^1^H^*j*^–^1^H^*k*^ pair with an internuclear distance *r*_*mn*_^*jk*^ is given by
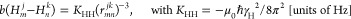
16

**Figure 7:**
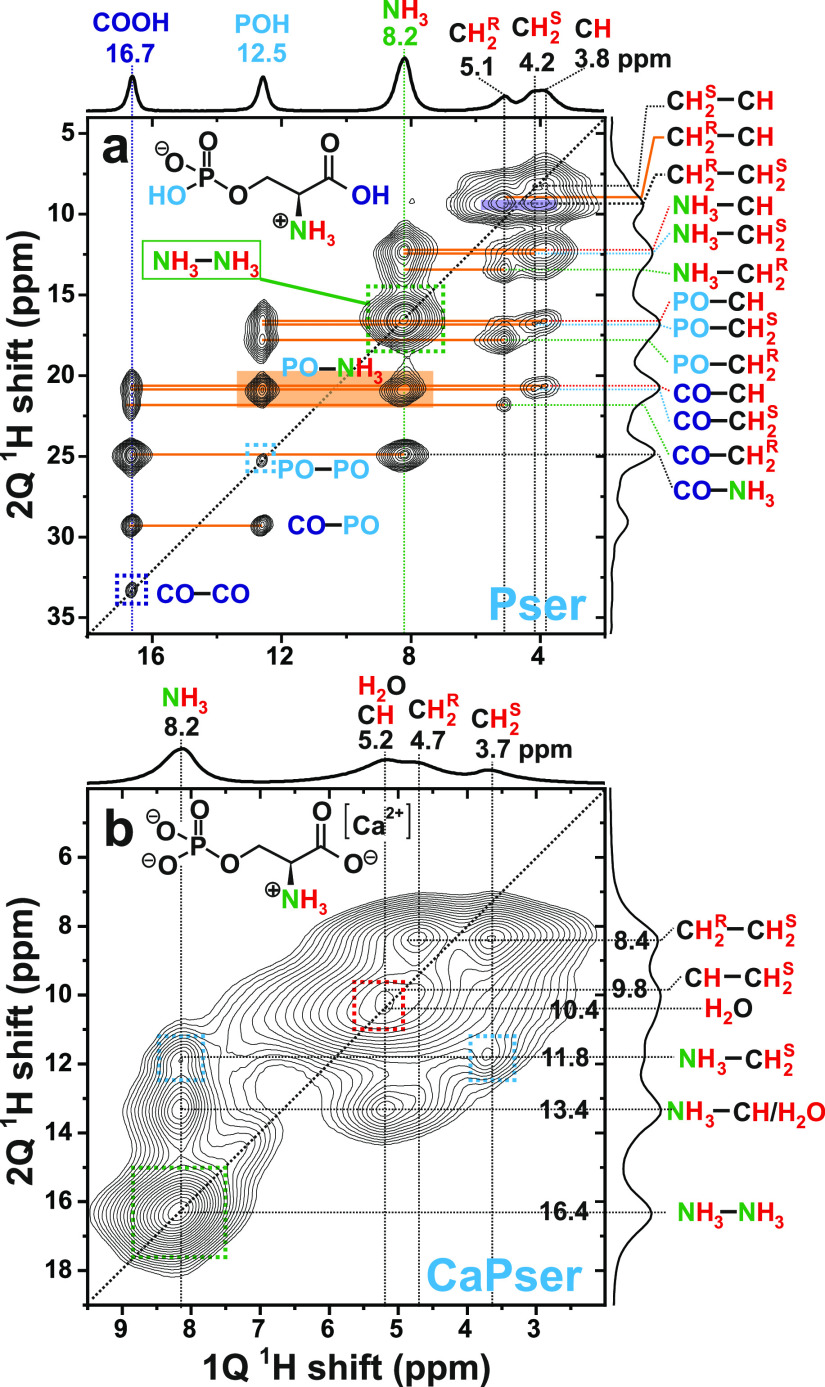
2Q–1Q ^1^H correlation NMR spectra recorded at 14.1 T and 66.00 kHz MAS from the (a) Pser and (b) CaPser samples by applying the BaBa recoupling sequence^58^ for τ_r_ = 15.15 μs to excite/reconvert 2QC. Each 2D NMR spectrum is shown together with projections along its horizontal 1Q (top) and vertical 2Q (right) dimensions. Note that the spectrum for CaPser is plotted over narrower shift ranges than that of Pser. In (a), the orange and blue areas mark the PO**H**···N**H**_3_^+^ and C**H**^R^H···CH**H**^S^ pairs of correlation peaks, respectively, while the green square encloses the N**H**_3_^+^ autocorrelation signal. In (b), the green and red squares indicate the autocorrelation peaks from the N**H**_3_^+^and **H**_2_O moieties, respectively, whereas the two N**H**_3_^+^···CH**H**^S^ correlation peaks are enclosed by cyan squares.

Figure 7 displays 2Q–1Q ^1^H correlation NMR spectra recorded from Pser and CaPser by employing a very short 2QC excitation interval of τ_exc_ = 15 μs to ensure a quantitative relationship between the integrated 2D NMR peak intensities and the squared effective dipolar coupling constant for the various ^1^H–^1^H pairs (Section 3.4), while also offering valuable ^1^H NMR peak assignments. Indeed, besides the well-separated and by fast-MAS readily resolved ^1^H resonances of the COO**H**, PO**H**, and N**H**_3_ moieties of Pser shown in Figure 2b (that were first assigned in ref (35)), the *precise*^1^H chemical shifts of the aliphatic protons listed in Table 1 were obtained from the 2Q–1Q ^1^H correlation NMR spectra of Figure 7.

Two ^1^H^*j*^ and ^1^H^*k*^ sites with the respective 1Q shifts δ_1Q_ = δ_H_^*j*^ and δ_1Q_ = δ_H_^*k*^ produce two 2Q–1Q correlation NMR peaks at the shift pairs {δ_2Q_, δ_1Q_} = {δ_H_^*j*^ + δ_H_^*k*^, δ_H_^*j*^} and {δ_2Q_, δ_1Q_} = {δ_H_^*j*^ + δ_H_^*k*^, δ_H_^*k*^}, where the 1Q and 2Q shifts (δ_2Q_) are encoded along the horizontal (direct) and vertical (indirect) dimensions of the 2D NMR spectrum, respectively.^36−40^ Hence, two spatially proximate but (magnetically) *in*equivalent ^1^H sites yield two 2Q–1Q correlation NMR signals, whereas two nearby equivalent protons produce one sole 2D NMR peak at {δ_2Q_, δ_1Q_} = {2δ_H_^*j*^, δ_H_^*j*^}; such an “autocorrelation” peak appears aligned with the “diagonal” of the 2D NMR spectrum, as indicated by the dotted line in Figure 7. The {δ_2Q_, δ_1Q_} coordinates for the various proton pairs of Figure 7 are collected in Table S5.

We remind that although all ^1^H sites are crystallographically inequivalent in both PSer/CaPser structures, the rapid rotational motion of the NH_3_^+^ moiety around the C^α^–N axis renders all amino protons equivalent. They produce a strong autocorrelation peak at {δ_2Q_, δ_1Q_} = {16.4, 8.2} ppm: consistent with their overall shortest ^1^H–^1^H distances accompanied by the highest multiplicity of the contributing couplings (Tables S2/S3), this 2Q–1Q correlation signal is the overall strongest in the 2D NMR spectra from both the Pser and CaPser structures (Figure 7), despite that the rapid molecular dynamics reduces the effective ^1^H–^1^H dipolar interaction of the amino moiety by 1/2 (which was also accounted for in our *b*_eff_^2^ calculations). Although the rotational motion of the N**H**_3_^+^ group is also expected to weaken the through-space interactions to protons of neighboring groups, the quantitative H^*j*^–H^*k*^ distance analysis presented in Section 3.8 did not reveal any such effects (as for the P–H distances discussed in Section 3.5).

Besides the dipolar contacts within the amino moiety, the protons of the methylene group of both Pser/CaPser molecules—and the **H**_2_O sites in the case of CaPser—account for the second largest ^1^H–^1^H dipolar interactions (Tables S2/S3). This is confirmed by the strong C**H**^R^H···CH**H**^S^ correlation peaks with δ_2Q_ = 9.3 ppm and δ_1Q_ = {5.1, 4.2} ppm observed from Pser (Figure 7a) and δ_2Q_ = 8.4 ppm along with δ_1Q_ = {3.7, 4.7} ppm for CaPser (Figure 7b). Notably, these inferences from the 2Q–1Q correlation NMR spectra fully corroborate the finding of distinct chemical shifts of the two methylene protons (Table 1), while the 2Q–1Q NMR spectra of Figure 7 offer a superior NMR-signal resolution relative to the ^1^H MAS NMR counterparts of Figure 2b, thereby greatly improving the accuracy of the ^1^H chemical shift of the aliphatic groups. Moreover, given the markedly longer intermolecular C**H**···C**H** distance (Tables S2/S3), the autocorrelation peak observed from CaPser at {δ_2Q_, δ_1Q_} = {10.4, 5.2} ppm in Figure 7b stems mainly from the ^1^**H**_2_O molecule of CaPser.

Notably, the H-bonding of the protons of the phosphate/carboxy groups between neighboring Pser molecules results in two significant 2Q–1Q correlation peaks at δ_2Q_ = 23.3 ppm and δ_1Q_ = {16.6, 12.6} ppm in Figure 7a. These 2D NMR signal intensities are markedly higher than the two weak autocorrelation peaks at the {δ_2Q_, δ_1Q_} shift pairs of {33.3, 16.6} ppm and {25.3, 12.6} ppm (Figure 7a) that reflect the respective intermolecular COO**H**···COO**H** and PO**H**···PO**H** contacts, as expected from their significantly longer ^1^H–^1^H distances in Table 3 (also see Figure 6).

**Table 3:** Effective ^1^H–^1^H Distances of Pser^a^

sites	*f*_NMR_	*f*_DFT_	*f*_XRD_	*r*_eff_^NMR^(*r*_eff_^DFT^) (pm)	Δ*r*^DFT^ (pm)	*r*_eff_^NMR^(*r*_eff_^XRD^) (pm)	Δ*r*^XRD^ (pm)
COO**H**							
COO**H**	0.005	0.003	0.003	296(331)	–35	288(331)	–43
PO**H**	0.028	0.020	0.014	254(267)	–13	247(277)	–30
N**H**_3_	0.159	0.209	0.173	202(194)	8	197(196)	1
C**H**^R^H^S^	0.019	0.016	0.020	270(277)	–7	263(263)	0
CH^R^**H**^S^	0.019	0.021	0.013	240(236)	4	234(252)	–18
C**H**	0.015	0.014	0.020	282(284)	–2	275(261)	14
PO**H**							
PO**H**	0.004	0.003	0.003	305(333)	–28	297(334)	–37
N**H**_3_	0.096	0.085	0.050	220(225)	–5	214(241)	–27
C**H**^R^H^S^	0.084	0.074	0.060	188(192)	–4	183(194)	–11
CH^R^**H**^S^	0.025	0.020	0.019	257(268)	–11	250(265)	–15
C**H**	0.024	0.020	0.018	230(238)	–8	224(237)	–13
N**H**_3_							
N**H**_3_							
C**H**^R^H^S^	0.094	0.062	0.054	221(237)	–16	215(238)	–23
CH^R^**H**^S^	0.129	0.110	0.101	210(216)	–6	204(214)	–10
C**H**	0.112	0.183	0.306	215(198)	17	209(178)	31
C**H**^R^H^S^							
C**H**^R^H^S^	n.d.^b^	0.002	0.002	(421)		(423)	
CH^R^**H**^S^							
C**H**	0.091	0.077	0.061	185(191)	–6	180(194)	–14
CH^R^**H**^S^							
CH^R^**H**^S^	n.d.^b^	0.003	0.002	(375)		(374)	
C**H**	0.096	0.083	0.085	206(212)	–6	200(206)	–6
C**H**							
C**H**	n.d.^b^	0.003	0.002	(373)		(377)	

a Effective ^1^H–^1^H distances *r*_eff_^X^ and fractional dipolar contacts *f*_X_ for X = {NMR, DFT, XRD}. The uncertainties (±1σ) of all {*f*_DFT_, *f*_XRD_} and {*r*_eff_^DFT^, *r*_eff_^XRD^} values are ±4 and ±0.7%, respectively. The corresponding uncertainties of the heavily overlapping 2Q–1Q correlation signals from the various aliphatic protons are σ(*f*_NMR_) = ±10% and σ(*r*_eff_^NMR^) = ±3%, whereas lower uncertainties of σ(*f*_NMR_) = ±2.5% and σ(*r*_eff_^NMR^) = ±0.9% apply for all other H^*j*^–H^*k*^ pairs that yielded well-resolved 2D NMR correlation peaks. The effective ^1^H–^1^H distances {*r*_eff_^NMR^, *r*_eff_^DFT^, *r*_eff_^XRD^} were calculated from eqs 10, 11, 17, and 18. The *f*_NMR_(N**H**_3_–N**H**_3_) and *f*_NMR_(C**H**^R^H^S^–CH^R^**H**^S^) data were obvious outliers and were excluded from the analysis. See Table 2 for further details.

b The fractional dipolar contact is negligible and could not be determined by NMR. This value was consequently omitted in the normalization of the {*f*_DFT_, *f*_XRD_} data sets to a unity sum.

### 3.8 Homonuclear ^1^H–^1^H Distance Determinations

By utilizing the procedure outlined in Section 3.4, the integrated 2D NMR intensities of the 2Q–1Q correlation spectrum recorded from Pser (Figure 7a) were used to derive the sets of {*f*_NMR_(H^*j*^–H^*k*^)} and {*r*_eff_(H^*j*^–H^*k*^)} results for the various H^*j*^–H^*k*^ pairs, where
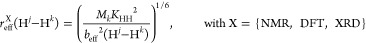
17Within a trivial change of notation S^*j*^ → H^*j*^, all expressions are identical to those of Section 3.4, except for the calculation of *b*_eff_^2^(H^*j*^–H^*k*^), which depends on whether the two proton sites are equivalent in the presence of molecular dynamics
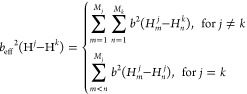
18Our effective-distance analysis included all non-negligible H_*m*_^*j*^–H_*n*_^*k*^ dipolar interactions but those within the methylene and amino moieties, which incidentally manifested the overall strongest dipolar contacts (Section 3.7) but which for unknown reasons produced markedly higher 2D NMR intensities than expected (note that violation of eq 14 cannot explain these findings because it would merely result in *underestimated* 2Q–1Q NMR peak intensities).

Table 3 lists the NMR-derived results along with the data from the “XRD” and “DFT” structures of Pser. With the COO**H**···N**H**_3_^+^ and COO**H**···C**H**^R^H distances as sole exceptions, a consistently better agreement is observed between the NMR experiments and the DFT structure relative to its XRD counterpart, as mirrored in the respective correlation coefficients *R*^2^ = 0.908 and *R*^2^ = 0.784. The improvements are particularly evident for the effective COO**H**···CH**H**^S^ and N**H**_3_^+^···C**H** distances as well as for the intermolecular COO**H**···PO**H** distance, where the difference *r*_eff_^NMR^–*r*_eff_^DFT^ = −13 pm is markedly lower than that of *r*_eff_^NMR^–*r*_eff_^XRD^ = −30 pm; see Figure 6 and Table 3. However, although most of the remaining deviations between the NMR- and DFT-derived distances remain within the experimental/computational uncertainties, it is notable that all experimentally obtained effective COO**H**···PO**H**, COO**H**···COO**H**, and PO**H**···PO**H** distances are consistently shorter than their counterparts in the DFT—and notably the unrefined XRD—structures, suggesting that the precise intermolecular H-bond lengths may be slightly overestimated even in the refined structure; see Figure S4c,d.

We did not pursue a quantitative analysis of the 2Q–1Q correlation NMR spectrum from CaPser (Figure 7b), whose strongly overlapping signals from the aliphatic protons and those of the water molecule would lead to significant uncertainties. Although the resolution is slightly better in the 2D NMR spectrum obtained at a longer excitation period of 61 μs (Figure S2b), an accurate/reliable distance analysis is hampered by the violation of eq 14. Nonetheless, the result obtained from the 2Q–1Q NMR counterpart recorded from Pser with τ_exc_ = 61 μs (Figure S2a) overall corroborated the results of Table 3, whereas as expected, the 2D NMR intensities associated with the weakest dipolar contacts were comparatively emphasized (see Section 3.4.1), thereby leading to underestimated *r*_eff_^NMR^(H^*j*^–H^*k*^) values (data not shown).

## 4 Discussion

### 4.1 Features of the Dipolar-Based Distance Determination Protocol

We confirmed the improved accuracies of the DFT-refined Pser and CaPser structures by applying a straightforward powder NMR-based distance-analysis protocol introduced by Yu et al.,^42^ which was successfully applied to validate a diffraction-derived structure of monetite, CaHPO_4_. Herein, we demonstrated that the procedure is also readily applicable to organic structures with significantly stronger effective ^1^H–^1^H and ^31^P–^1^H dipolar coupling constants, encompassing widely spanning ^1^H_*m*_^*j*^–^1^H_*n*_^*k*^ interaction strengths, thereby presenting markedly more challenging cases for meeting the requirements that enable quantitative analyses of the 2D NMR data (Section 3.4). The accuracies of the DFT-refined structures were further corroborated by the GIPAW-generated ^1^H and ^13^C chemical-shift constraints. Although we employ the more specific S^*j*^–H^*k*^ (S = {^1^H, ^13^C, ^31^P}) notation, the general procedure for extracting effective dipolar-coupling constants/distances by hetero- or homo-nuclear MAS NMR is applicable to any S^*j*^–I^*k*^ or S^*j*^–S^*k*^ spin pair. The protocol may be summarized by the following steps:

(1) Record a 2D NMR spectrum in the regime where its integrated peak intensities relate quantitatively to *b*_eff_^2^(S^*j*^–H^*k*^) (eq 14), where S^*j*^ may constitute ^1^H or a distinct spin species. Notably, while we employed CP/HETCOR and homonuclear 2Q dipolar recoupling, any homo-/hetero-nuclear polarization-transfer NMR scheme may be utilized,^36−40^ such as dipolar-driven hetero-nuclear multiple-quantum coherence (HMQC).^69,98,99^ Although ^1^H spin diffusion remained very limited during the short CP contact periods of our HETCOR experiments at very fast MAS, active ^1^H–^1^H decoupling during the CP stage may be beneficial for improving the accuracy of the distance analysis.
(2) Deduce the set {*f*_NMR_(S^*j*^–H^*k*^)} by feeding the integrated 2D NMR peak intensities into eq 7.
(3) Obtain the corresponding set {*f*_model_(S^*j*^–H^*k*^)} from the atom coordinates of the structure “model” to be evaluated. Herein, we employed XRD-derived structures before and after refinements by DFT, but the model structure may originate from any experimental or crystal structure prediction (CSP) approach, such as molecular modeling; for example, see refs and (48) and (100–104).
(4) To assess the validity of the structure model, the agreement between {*f*_NMR_(S^*j*^–H^*k*^)} and {*f*_model_(S^*j*^–H^*k*^)} may either be contrasted directly or by evaluating the corresponding effective-distance {*r*_eff_^NMR^(S^*j*^–H^*k*^)} and {*r*_eff_^model^(S^*j*^–H^*k*^)} data from eqs 12 and 17.

Notably, the straightforward and rapid calculations of steps (3) and (4) enable validations of a vast number of potential model structures, as in relatively few previous NMR crystallography implementations.^100,105,106^ However, access to the set of effective S^*j*^–H^*k*^ distances (encompassing H^*j*^–H^*k*^) from the {*f*_NMR_(S^*j*^–H^*k*^)} set requires some information that is not available from the single 2D NMR spectrum. It is well known^36−39^ that eqs 11 and 15 imply that once *one* effective S^*j*^–H^*k*^ distance is known, then those of all other S^*j*^–H^*k*^ pairs are readily calculated from the set of fractional intensities {*f*_NMR_(S^*j*^–H^*k*^)} obtained from the 2D NMR spectrum. However, besides our very recent work,^42^ we are unaware of direct applications of this simple property for the purpose of deriving (near)-complete sets of internuclear distances from one 2D NMR spectrum alone. Moreover, rather than biasing the effective-distance analysis on one sole S^*j*^–H^*k*^ value, it is more accurate to exploit the total effective squared effective dipolar coupling constant [*b*_eff_^2^(tot); eq 9] for the {*f*_NMR_(S^*j*^–H^*k*^)} → {*r*_eff_^NMR^(S^*j*^–H^*k*^)} mapping.^42^ Hence, equating *b*_eff_^2^(tot) from eq 11 with that of the model structure (eq 10) results in direct assessments of all effective distances of the model [or rather, all spin pairs for which *f*_NMR_(S^*j*^–H^*k*^) data are available] by, for instance, evaluating their rmsd to the NMR counterparts.

The inherent multispin nature of all crystal structures underscores the relevance of the squared effective dipolar coupling constants, *b*_eff_^2^(S^*j*^–H^*k*^) and *b*_eff_^2^(H^*j*^–H^*k*^) (eqs 8 and 18), as the relevant parameters: only *those* are directly proportional to the integrated 2D NMR intensities of eqs 11 and 15 [*not* the individual spin-pair constants *b*^2^(S_*m*_^*j*^–H_*n*_^*k*^) and *b*^2^(H_*m*_^*j*^–H_*n*_^*k*^) of eqs 6 and 16]. Hence, any internuclear distance measurement involving protons in organic structures must account for dipolar interactions over several Å in the structure analysis (i.e., also intermolecular interactions^100,107,108^), which applies even for ^13^C–^13^C distance determinations at natural abundance.^103^ Since the effective dipolar interactions/distances are in general not readily obtained by current NMR methods and must be calculated from the atom coordinates of some structure model, these multispin effects underpin the current absence of *de novo* measurements of entire sets of internuclear distances from powders by NMR alone.

#### 4.1.1 Previous NMR Crystallography Approaches

Relative to previous dipolar-interaction-based NMR crystallography procedures, the interatomic effective-distance extraction procedure introduced in ref (42), and extended and discussed further herein, features a unique combination of *minimal* efforts in *both the generation of experimental NMR data and the analysis thereof* (here, we disregard the present and previous chemical-shift-based assessments of (new) structure models, which are nowadays made routinely^45−48,80^). With some notable exceptions commented on below, all hitherto employed hetero- and homonuclear dipolar-based NMR crystallography studies relied on monitoring the NMR-signal buildup from dipolar-driven polarization transfers and/or that of 2QC for progressively increasing dipolar recoupling periods,^36−38,92,100−110^ where an adequate sampling of the spin dynamics necessitates the recording of several 2D NMR spectra and the subsequent integration of all relevant peak intensities. Nonetheless, as shown herein, all these 2D NMR spectra are redundant but that recorded with a very short dipolar recoupling time period. Unfortunately, that experiment is the most time-consuming one to arrange due to the low spectral S/N ratio relative to those recorded with near-maximum 2D NMR peak intensities.

The subsequent *NMR-data analysis* of the set of 2D NMR-signal buildup data from previous NMR crystallography protocols is often even more effort-intensive than the experiments. While being less cumbersome for heteronuclear NMR experiments (owing to the commutation of the spin operators among distinct heteronuclear dipolar interactions^36,71,74^), the strong ^1^H–^1^H interactions and their associated spin-diffusion processes in organic/biological samples anyway require explicit considerations of multispin systems (*vide supra*) for anything beyond qualitative experimental constraints; this becomes mandatory for distance analyses of strongly coupled homonuclear systems (such as for protons) where the lack of analytical solutions of the spin dynamics of 2QC/polarization-transfer NMR data typically requires recourse to numerically exact spin dynamics simulations,^75,111,112^ which become prohibitively time-consuming for iterative-fitting purposes with more than 3–4 coupled spins-1/2. Indeed, current dipolar-based NMR crystallography analyses of multispin systems that invoked numerical simulations were restricted to one of the following options:

(i) Using the atomic coordinates of a known structure model to calculate the {*b*(S_*m*_^*j*^–S_*n*_^*k*^)} set subsequently utilized in the multiple-spin-dynamics simulations,^37,92,103,108,109^ whose extremely time-consuming computations were necessarily restricted to *validating one* structure model against the experimental results. (ii) The (large) spin system was approximated by a smaller counterpart of 2–4 spins,^102,104,109,110^ with the internuclear distances varied to fit the NMR-signal buildup to the experimental counterpart. Such internuclear-distance extraction procedures have been applied extensively to “isolated” spin pairs, such as ^13^C–^13^C pairs in organic/biological samples,^36,38,113,114^ where specific ^13^C-site labeling in conjunction with diluting the ^13^C–^13^C pairs by co-crystallization with a natural abundance material is often straightforward.^36,38^ Alternatively, selective dipolar recoupling specific to one spin pair may be employed.^115−117^

Another internuclear-distance analysis strategy does not involve the direct monitoring of the dependence of the 2QC or polarization-transfer amplitudes for increasing dipolar recoupling periods but merely targets either the (slow) MAS ^1^H NMR spectrum^37,118^ or the rotor-encoded 2QC spinning sideband formation in a 2Q–1Q correlation NMR spectrum recorded with Δ*t*_1_ = τ_r_/*N*.^37,119,120^ While being fairly effortless experimentally, the subsequent numerical analyses are equally time-/computer-intensive as the NMR-signal buildup approaches discussed above. Hence, all hitherto presented work based on rotor-encoded 2Q spinning sidebands from 3D crystal structures invoked approximations to avoid prohibitive calculations of large spin systems, such as the summation of numerically exact simulations of a large number of NMR spectra from *as-assumed* isolated pairs of half-integer spins.^121,122^ Although such approximative analysis procedures have had some success in validating single structure models, they require assumptions that are difficult to justify rigorously; see refs (121)–^123^ for details.

The NMR crystallography protocol applied herein is unique. However, out of the currently existing options, it shares many of the favorable properties of the ^1^H spin-diffusion-based approach by Emsley and co-workers,^100,106,107^ which also offers structural validations of large ^1^H–^1^H distance sets with comparatively low investments in experimental and computational time/efforts, stemming from a phenomenological (and thereby approximate) analysis of the cross-peak signal buildup in a series of 2D NMR spectra with increasing mixing periods.

#### 4.1.2 Practical Limitations

One limitation of our NMR experimentation, which indeed precluded analysis of some 2D NMR signals, concerns the compromised ^1^H spectral resolution by the absence of explicit ^1^H–^1^H decoupling. All our 2D NMR experiments involved very fast MAS (ν_r_ = 66 kHz) that yielded sufficient discrimination of most ^1^H resonances yet not for analyzing the 2Q–1Q correlation ^1^H NMR experiment from CaPser (Figure 7b). Improved spectral resolution is often arranged by either employing ^1^H–^1^H decoupling^91,92,100,105−107^ or probing the proton dynamics via ^13^C detection.^105,108^ While homonuclear proton decoupling is readily implemented during both evolution periods of a 2Q–1Q ^1^H NMR experiment, current decoupling techniques are developed for slower-MAS applications and their performance deteriorate at fast MAS. Reducing the spinning speed effectively lengthens the available minimum dipolar recoupling period (Section 2.2), which may compromise the adherence to eq 14. Hence, although our internuclear-distance analysis is formally independent on both the MAS rate and the specific recoupling method employed, for strongly coupled systems/samples, the fundamental requirement of operating in the limit of eq 14 is strict.

However, the ^1^H spectral resolution improves for increasing MAS rate, while the minimum duration of the dipolar recoupling sequence shortens (whose period is a multiple of τ_r_; Section 2.2). Hence, implementations at progressively faster MAS relieve both these practical limitations and improve the accuracy of the internuclear distance analysis.

### 4.2 Discussion on Anisotropic ^31^P Chemical Shifts

Table 4 lists the chemical-shift anisotropy and asymmetry parameter of the ^31^P site in each Pser and CaPser molecule, as obtained by either experiments or DFT/GIPAW calculations. For Pser, the DFT-derived {δ_aniso_; η} = {−55.2 ppm; 1.00} pair is in excellent agreement with the {−56.5 ppm; 0.91} counterparts obtained by iterative fitting of a slow-MAS ^31^P NMR spectrum (Figure S3a). Our results may also be contrasted with the following previously reported {δ_aniso_; η} data obtained by MAS NMR: {−56.3 ppm; 0.83},^35^ {−57 ppm; 0.9},^50^ and {|δ_aniso_| = 55.3 ppm; 0.91}^51^ (where only the magnitude of the anisotropy was reported in ref (51)). Next, considering the ^31^P CSA parameters of CaPser in Table 4, the DFT/GIPAW-derived {δ_aniso_; η} pair of {71.3 ppm; 0.32} accords very well with that from NMR (Figure S3b), {67.2 ppm; 0.33}. The latter data even coincide with those of |δ_aniso_| = 68 ± 1 ppm and η = 0.33 reported by Greenwood et al.,^51^ whereas the sole other ^31^P CSA study reported {δ_aniso_; η} = {71.3 ppm; 0.46}.^25^ For both Pser and CaPser, we conclude that the DFT/GIPAW calculations successfully reproduced the experimental CSA parameters.

**Table 4:** DFT/GIPAW-Derived ^31^P and ^13^C CSA Parameters^a^

	Pser					CaPser				
site	δ_aniso_ (ppm)	η	δ_*xx*_ (ppm)	δ_*yy*_ (ppm)	δ_*zz*_ (ppm)	δ_aniso_ (ppm)	η	δ_*xx*_ (ppm)	δ_*yy*_ (ppm)	δ_*zz*_ (ppm)
^31^**P**	–55.2	1.00	55.1	0.1	–55.2	71.3	0.32	71.3	–24.4	–47.1
	(−56.5)	(0.91)				(67.2)	(0.33)			
^13^**C**OOH	86.9	0.47	105.7	146.4	256.4	–67.9	0.76	231.2	179.9	103.7
^13^**C**H_2_	–35.9	0.09	85.8	82.7	30.4	–38.5	0.54	95.8	74.9	27.6
^13^**C**H	–16.7	0.85	70.3	56.2	38.2	–18.1	0.29	68.2	63.1	38.6

a DFT-derived ^31^P and ^13^C CSA parameters presented together with ^31^P MAS NMR-determined δ_aniso_ and η values (in parentheses).

We proceed by making some further remarks concerning the prospect of using the ^31^P CSA parameters as a profiling tool for the very first bone mineralization, as proposed by Wu et al.^25^ They employed the shift “span” parameter Ω = |δ_*zz*_ – δ_*xx*_| to specify the CSA magnitude,^72^ whereas their primarily targeted *sign* of the anisotropy was encoded by the parameter ι = δ_iso_ – δ_*yy*_, which was found to be negative for ^31^**P**O_4_^2–^ sites devoid of Ca^2+^ contacts but positive for ^31^**P**O_4_^2–^···Ca^2+^ environments.^25^ Notably, the sign of ι always matches that of δ_aniso_ defined by eq 4, which captures both information from Ω and ι in one single parameter. We moreover highlight the following:

(i) The sign reversal of δ_aniso_ (or the “ι” parameter^25^) from negative to positive values is not a feature of PO_4_^2–^···Ca^2+^ contacts per se, as is evident from the similar trends of ^31^P CSA values of the ^31^PO_4_ moiety of Pser for variable pH^27^ as well as from analogous sign-reversal effects on δ_aniso_ reported for H_*n*_PO_4_^(3–*n*)–^ groups of inorganic phosphate phases.^124^ Indeed, the δ_aniso_ values of the respective PO_2_O^–^(OH) and PO_2_(O^–^)_2_ groups of Pser and CaPser follow the same sign trend for a OH → O^–^ conversion as their inorganic phosphate counterparts,^124^ although both ^31^P sites of Pser and CaPser feature a ≈40% lower CSA magnitude. Odd/even sign alterations of δ_aniso_ are also observed for the ^13^**C**OOH group of Pser and its ^13^**C**OO^–^ counterpart of CaPser in Table 4 (as well as for other amino acids^79^). In all above-mentioned cases, protons act as the charge-balancing species, but the same ^31^P CSA effects hold for Ca^2+^, Na^+^, or other cation species.^25,27,124^

(ii) It follows from the remarks made in (i) that a sign reversal of the ^31^P chemical-shift anisotropy does not necessarily imply the emergence of PO_4_^2–^···Ca^2+^ motifs (at a phosphorylated NCP residue),^25^ but such ±δ_aniso_ effects may also stem from local pH alterations of the surrounding body fluid. Hence, although we believe that the inference of Wu et al.^25^ concerning the diagnostics of the sign of δ_aniso_/ι as implying (the absence of) PO_4_^2–^···Ca^2+^ contacts is likely correct, further studies are warranted for a strict consolidation of this equivalence, a topic that has not received due attention in the community that intersects the NMR and biomineralization fields.

### 4.3 Discussion on Anisotropic ^13^C Chemical Shifts

The DFT/GIPAW-derived chemical-shift tensor parameters are listed in Table 4 for each ^13^**C**H, ^13^**C**H_2_, and ^13^**C**OO(H) site of Pser and CaPser. We did not obtain experimental ^13^C CSA values, and we are not aware of experimental data neither on the aliphatic sites of Pser nor for any ^13^C site of polycrystalline CaPser (yet see ref (27)). There is to our knowledge only one previous study on the ^13^**C**OOH chemical-shift parameters of polycrystalline Pser, where Potrzebowski et al.^35^ reported the following values: {δ_*xx*_, δ_*yy*_, δ_*zz*_} = {111, 152, 247} ppm and {δ_iso_; δ_aniso_; η} = {171.0 ppm; 76 ppm; 0.54}. The CSA data predicted by the DFT/GIPAW calculations (Table 4) agree reasonably well with the experimental results,^35^ and they match the latter markedly better than the DFT calculations with the gauge independent atomic orbital (GIAO) method^125^ employed by Potrzebowski et al. in 2003,^35^ which primarily reflects the general advances in DFT computations and the improved accuracy offered by plane-wave methods for periodic structures.

Noting that the chemical-shift parameters of the carboxy group of Pser have been examined in detail for variable protonation states of the molecule,^27,35^ we onward focus on the DFT/GIPAW-predicted alterations of the principal values and {δ_aniso_; η} parameters of the ^13^**C**OOH and ^13^**C**OO^–^ sites of Pser and CaPser in relation to well-established general ^13^C CSA-parameter trends upon a COO^–^ → COOH conversion:^27,35,77−79^ (i) the δ_*yy*_ value decreases from 180 ppm (^13^**C**OO^–^ of CaPser) to 146 ppm (^13^**C**OOH of Pser), accompanied by (ii) an increased shift of the most deshielded tensor element from 231 ppm to 256 ppm (Table 4). (iii) The most shielded tensor component remains essentially unaffected by the protonation,^27,35,77−79^ as witnessed by the values δ_*xx*_ = 106 ppm (Pser) and δ_*zz*_ = 104 ppm (CaPser) in Table 4. Note that the exchanged δ_*xx*_/δ_*zz*_ components account for the previously commented sign reversal of δ_aniso_ (Section 4.2) via eqs 2 and 4. It is gratifying that our observed alterations in the {δ_*xx*_, δ_*yy*_, δ_*zz*_} values semiquantitatively reproduce the general experimental trends deduced by Gu and McDermott from a large set of amino acids.^79^

## 5 Conclusions

We have refined previously reported XRD-derived crystal structures of Pser and CaPser by DFT calculations and evaluated both the previous/refined structure options against experimental ^1^H, ^13^C, and ^31^P chemical shifts as well as direct {P–H^*k*^}, {C^*j*^–H^*k*^}, and {H^*j*^–H^*k*^} distance constraints, each extracted by one 2D MAS NMR experiment applied to the powdered sample with all isotopes at natural abundance. Whereas relatively modest improvements resulted for the XRD-derived structure of CaPser, much closer agreements with the NMR results were observed for the DFT-refined Pser structure.

A decisive advantage of our 2D NMR analysis strategy relative to previously reported NMR crystallography applications for obtaining internuclear distances is the minimum of efforts required both experimentally and numerically: provided that the various 2D correlation NMR signals are reasonably resolved (a limitation that applies to all 2D NMR-based analysis approaches to date) and that a sufficiently short dipolar recoupling period is employed such that eq 14 is obeyed, entire sets of effective interatomic distances are readily obtained from *one sole* 2D NMR experiment, without any fitting against phenomenological expressions or (typically) time-consuming multiple-spin simulations to reproduce the 2D NMR peak-intensity buildup for increasing dipolar recoupling periods, where each such data point demands the recording of one additional 2D NMR spectrum. Moreover, the application of numerically exact spin dynamics simulations remains, for practical reasons, model-dependent and may require knowledge of additional NMR parameters, such as anisotropic chemical shifts and/or dipolar/chemical-shift tensor orientations.

The price paid by our analysis procedure is that it only yields a set of “effective” S^*j*^–H^*k*^ or H^*j*^–H^*k*^ distances (each typically carries contributions from several dipolar interactions), in contrast with the entire sets of S^*j*^–H^*k*^ and H^*j*^–H^*k*^ distances in the crystal structure that is readily extracted by diffraction techniques but hitherto also not attainable by other NMR crystallography implementations to 3D structures of polycrystalline powders. We expect the comparatively rapid/effortless interatomic-distance assessments offered by the herein proposed ^1^H-based 2D MAS NMR analyses to be particularly useful for the pharmaceutical industry, where a readily obtained XRD-derived structure or a computer-generated model may be assessed in orders-of-magnitude less time/efforts than those of previous (yet more accurate) NMR analysis procedures, thereby offering a high-throughput screening of the validity of a given XRD/modeled structure, which if not deemed sufficiently accurate may be refined by DFT calculations and then reassessed against the 2D NMR results.

## Supporting Information Available

Additional NMR spectra (Figures S1–S3) and distance-correlation plots (Figure S4); DFT/GIPAW-derived chemical shifts of the Pser/CaPser structures before refinements (Table S1); interatomic distances in the Pser and CaPser structures before and after refinements (Tables S2 and S3); NMR-derived effective ^13^C–^1^H distances (Table S4); 2Q and 1Q ^1^H chemical-shift coordinates of the 2Q–1Q correlation NMR spectra (Table S5) (PDF)
