# Supplementary material for: Refined Structures of O-Phospho-l-serine and Its Calcium Salt by New Multinuclear Solid-State NMR Crystallography Methods
Source: J Phys Chem B. 2021 Sep 23;125(39):10985–1004. doi: 10.1021/acs.jpcb.1c05587 (PMC8503883; doi:10.1021/acs.jpcb.1c05587)
Supplement: Supplementary file 1 — jp1c05587_si_001.pdf [file jp1c05587_si_001.pdf]

Supporting Information for

Refined Structures of *O*-Phospho-L-serine and its Calcium Salt by New Multinuclear Solid-State NMR Crystallography Methods

Renny Mathew, Baltzar Stevensson, and Mattias Edén*

Department of Materials and Environmental Chemistry, Stockholm University, SE-106 91 Stockholm, Sweden

*Corresponding author. E-mail: *mattias.eden@mmk.su.se*

Contents

1. **Figure S1.** ^{13}C CPMAS NMR Spectrum of L-Serine.
2. **Figure S2.** Supplementary 2Q–1Q Correlation ^{1}H NMR Spectra.
3. **Figure S3.** Slow-MAS ^{31}P NMR Spectra of Pser and CaPser.
4. **Figure S4.** Correlation Plots of NMR/XRD/DFT Generated Interatomic Distances.
5. **Table S1.** Experimental and Calculated ^{1}H and ^{13}C Chemical Shifts From the XRD Structures Before Refinements.
6. **Table S2.** Shortest ^{31}P–^{1}H, ^{13}C–^{1}H, and ^{1}H–^{1}H Distances in the XRD-Derived Pser/CaPser Crystal Structures.
7. **Table S3.** Shortest ^{31}P–^{1}H, ^{13}C–^{1}H, and ^{1}H–^{1}H Distances in the Energy-Optimized Pser/CaPser Crystal Structures.
8. **Table S4.** Effective ^{13}C–^{1}H Distances.
9. **Table S5.** 2Q and 1Q ^{1}H Chemical Shift Coordinates.
10. **References.**

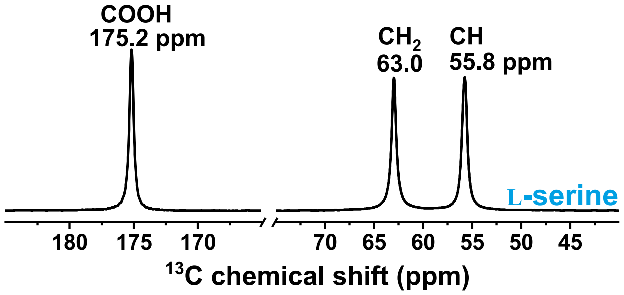

Fig. S1. ^{13}C CPMAS NMR spectrum recorded from a powder of L-serine under otherwise identical experimental conditions as in Fig. **2**a. The NMR spectrum is reproduced from the raw data presented by Mathew et al.,[S1] where we have corrected the NMR peak assignments of the CH and CH_2 groups that were unfortunately swapped in ref. S1.

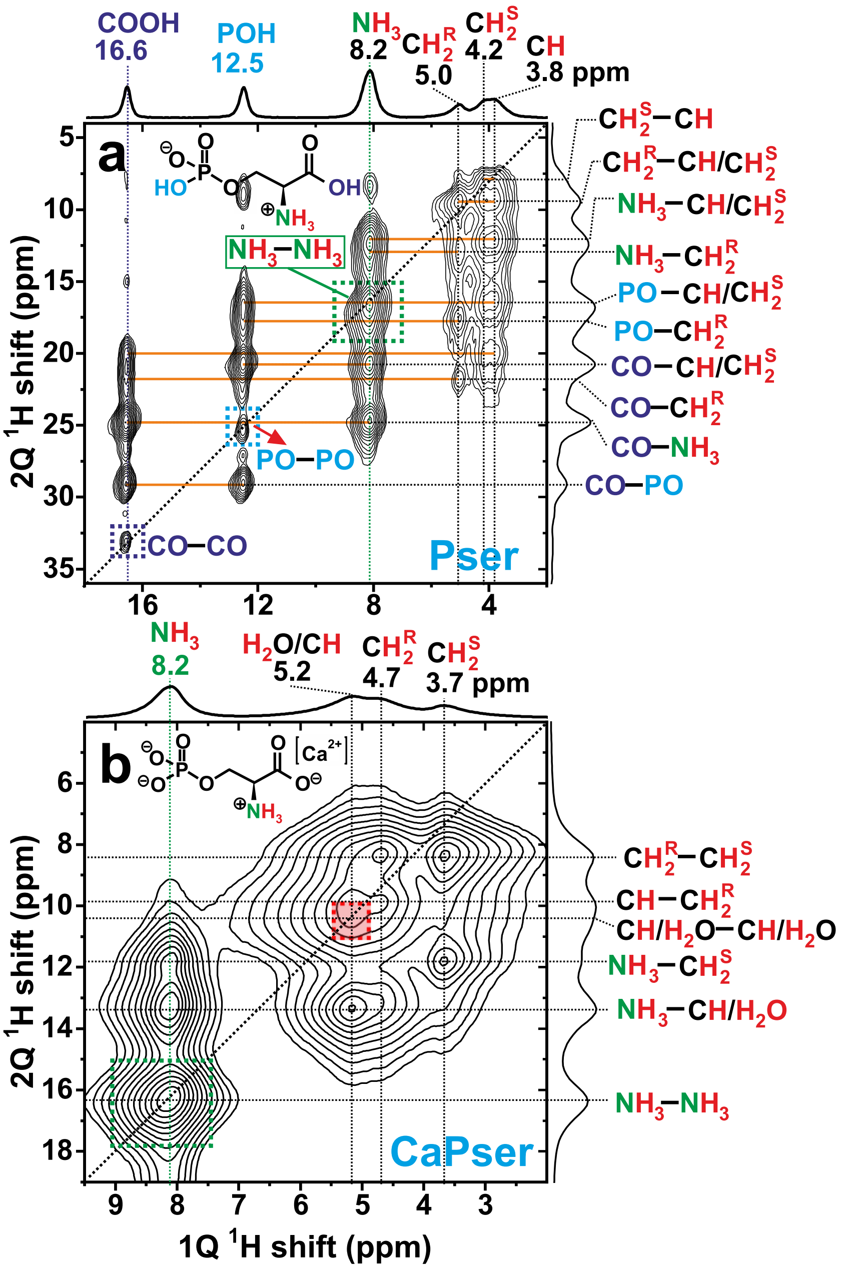

Fig. S2. 2Q–1Q ^{1}H correlation NMR spectra obtained from the (a) Pser and (b) CaPser at 14.1 T and 66.00 kHz MAS as in Fig. **7**, but instead utilizing the $[SR2_2^1]$ sequence[S2,S3] for 2QC excitation and reconversion: $\tau_{\text{exc}} = \tau_{\text{rec}} = 60.6$ μs. The brackets $[\cdots]$ mark the presence of two strong 90° pulses around the $R2_2^1R2_2^{-1}$ pulse block.[S2,S3] The green and red squares mark the autocorrelation signals of $N\mathbf{H}_3^+$ and $\mathbf{H}_2O$, respectively, where the latter overlap with minor $C\mathbf{H}\cdots C\mathbf{H}$ contributions. *Experimental Conditions:* The ^{1}H nutation frequency was $\nu_H = \nu_r/2$ =33.00 kHz during dipolar recoupling and 167 kHz for all strong 90°/180° pulses. A spin-echo of duration of $2\tau_r = 30.3$ μs was applied before the t_1-evolution interval. The 2D NMR acquisitions employed $\tau_{\text{relax}} = 1.5$ s and the following parameters: for Pser, $36(t_1)\times3000(t_2)$ time-points were acquired with dwell times of $\{\Delta t_1 = 2\tau_r;\ \Delta t_2 = 3.6\ \mu s\}$ and 768 accumulated transients/t_1-value; for CaPser, 75×850 time-points were acquired with dwell times of $\{\Delta t_1 = 3\tau_r;\ \Delta t_2 = \tau_r\}$ and 128 accumulated transients/t_1-value. The 2D data sets was zero-filled to 256×16384 (Pser) and 256×4096 (CaPser) points and apodized by a cos^2 and exponential (20 Hz for Pser and 5 Hz for CaPser) function along the indirect and direct dimensions, respectively.

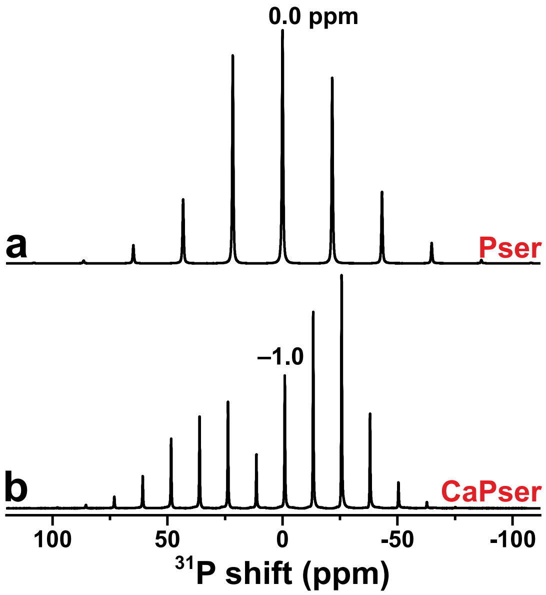

Fig. S3. ^{31}P MAS NMR spectra recorded from powders of (a) Pser and (b) CaPser by CPMAS and single pulses, respectively, under slow-MAS conditions at the corresponding rates ν_r = 2.00 kHz and ν_r = 3.50 kHz, respectively. The isotropic chemical shift at the centerband peak is marked in each spectrum. All other peaks are spinning sidebands.

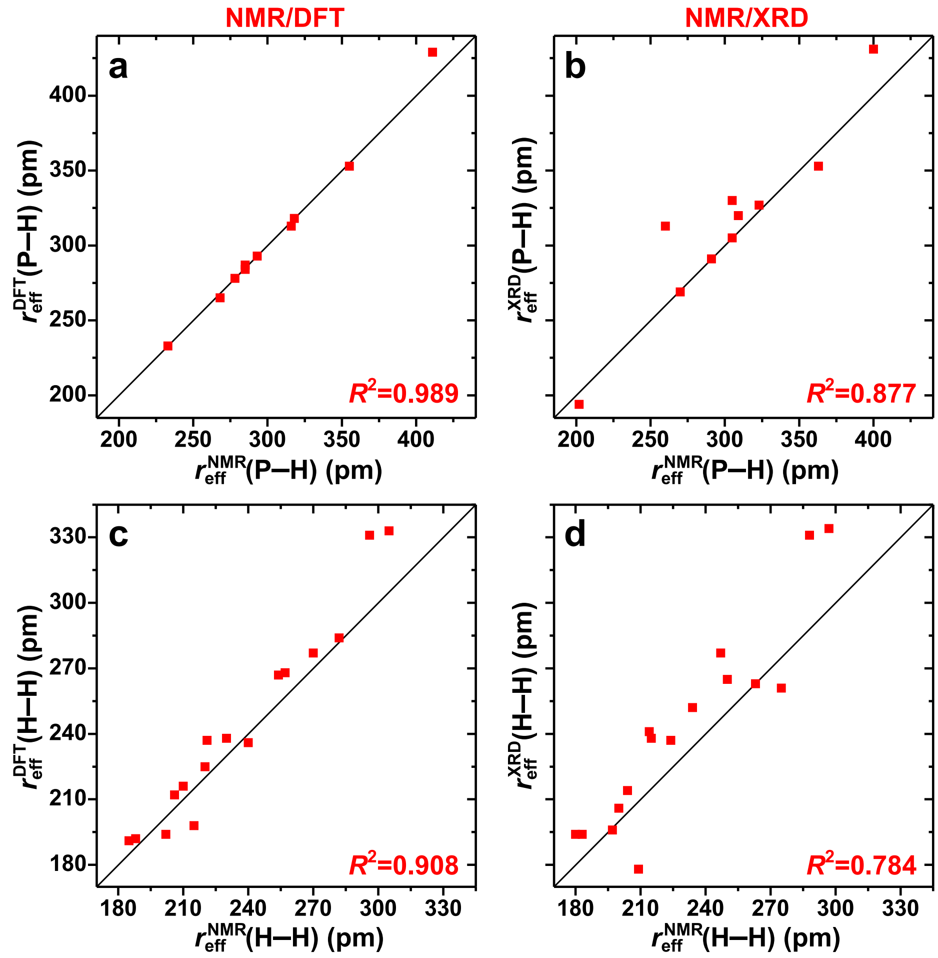

Fig. S4. Correlation plots of effective (a, b) P–H^k and (c, d) H^j–H^k internuclear distances obtained by XRD (a, c) after and (b, d) before structure refinements by DFT. The data plotted in (a, b) are those of Table **2**, as obtained from *both* the Pser and CaPser structures, whereas (c, d) only includes results from Pser (see Table **3**). Hence, the correlation coefficients in (a, b) may be contrasted with those reported in section **3.5** for each Pser and CaPser molecule.

Table S1. Experimental and Calculated ^{1}H and ^{13}C Chemical Shifts From the XRD Structures Before Refinements[a]

Site	Pser δ_{iso} (ppm)	CaPser δ_{iso} (ppm)
^{13}**C**		
**C**OOH	170.7(173.6)	174.0(173.9)
**C**H_2	64.2(69.1)	64.1(62.0)
**C**H	55.3(53.1)	56.9(53.3)
^{1}**H**		
COO**H**	16.7(2.2)	
PO**H**	12.5(1.8)	
N$\mathbf{H}_3$	8.2(3.0)	8.2(6.7)
C$\mathbf{H}^{R}$H	5.1(10.8)	4.7(7.2)
CH$\mathbf{H}^{S}$	4.2(7.5)	3.7(6.9)
C**H**	3.9(3.4)	5.2(5.0)
$\mathbf{H}_2$O		5.2(−3.0)

[a] Data as in Table **1**, but for chemical shifts calculated by DFT/GIPAW (values in parentheses) with the coordinates of the XRD-derived structures of Pser (ref. S4) and CaPser (ref. S5), *i.e.*, before their refinements by DFT.

Table S2. Shortest ^{31}P–^{1}H, ^{13}C–^{1}H, and ^{1}H–^{1}H Distances (in pm) in the XRD-Derived Pser/CaPser Crystal Structures[a]

	COO**H**	PO**H**	N**H**$_3$	C**H**^{R}H^{S}	CH^{R}**H**^{S}	C**H**	**H**$_2$O
Pser							
**P**	313	194	314;317;388	269	313;329	401;431;453;456	
**C**OOH	158	348	249;262;310	268	342;385	203	
**C**H$_2$	353;398	334	255;256;320	118	105	204	
**C**H	289	371	187;193;196	219	213	91	
COO**H**	461	364;380	230;266;324	315;448	354	311;443	
PO**H**		463	317;346;382	249	340;361	322	
N**H**$_3$			129;148;151	309;346;356	248;294;346	215;220;266	
C**H**^{R}H^{S}				586;594;594;619	189	249	
CH^{R}**H**^{S}					472;548	239;316	
C**H**						490;532	
CaPser							
**P**			300;339;362	291	350;356	310;311	297;305
**C**OOH			245;269;280	247	273;310	203	336;407
**C**H$_2$			247;275;336	102	107	201	355;368;371;405
**C**H			191;202;211	200	216	93	345;391
N**H**$_3$			136;147;160	335;341;362;364;391;406	236;312;327	223;239;264	259;317;328
C**H**^{R}H^{S}				553;553;574;574	168	227	297;312
CH^{R}**H**^{S}					553;553;574;574	289	285;340
C**H**						553;553;574;574	269;336
**H**$_2$O							127

[a] The as-indicated internuclear distances were used for calculating the respective f_{XRD} and $r_{\mathrm{eff}}^{\mathrm{XRD}}$ results of Tables **2**, **3**, and **S4**.

Table S3. Shortest ^{31}P–^{1}H, ^{13}C–^{1}H, and ^{1}H–^{1}H Distances (in pm) in the Energy-Optimized Pser/CaPser Crystal Structures[a]

	COO**H**	PO**H**	N$\mathbf{H}_3$	C$\mathbf{H}^{R}$H^{S}	CH$^{R}\mathbf{H}^{S}$	C**H**	$\mathbf{H}_2$O
Pser							
**P**	265	220	291;295	278	312;324	388;432;459;470	
**C**OOH	194	344	257;268;311	275	347;379	217	
**C**H_2	357;382	320	266;272;339	110	110	215	
**C**H	330	372	207;211;211	214	217	110	
COO**H**	456	334;398	240;240;326	354;407	315	365;417	
PO**H**		461	271;322;371	246	354;361	325	
N$\mathbf{H}_3$			166;169;173	303;347;367	246;309;368	243;250;297	
C$\mathbf{H}^{R}$H^{S}				556;597;601;631	179	246	
CH$^{R}\mathbf{H}^{S}$					477;544	252;299	
C**H**						476;538	
CaPser							
**P**			282;331;362	284	353;354	296	269;303
**C**OOH			236;262;280	263	272;312	215	332;407
**C**H_2			275;281;324	110	110	217	342;369;370;392
**C**H			208;210;215	214	216	109	333;392
N$\mathbf{H}_3$			167;167;172	349;352;374;380;384;395	256;300;319	234;241;300	260;297;323
C$\mathbf{H}^{R}$H^{S}				553;553;574;574	178	251	289;305
CH$^{R}\mathbf{H}^{S}$					553;553;574;574	307	284;320
C**H**						553;553;574;574	248;337
$\mathbf{H}_2$O							155

[a] The as-indicated internuclear distances were used for calculating the respective f_{DFT} and $r_{\mathrm{eff}}^{\mathrm{DFT}}$ results of Tables **2**, **3**, and **S4**.

Table S4. Effective ^{13}C–^{1}H Distances[a]

C–**H** pair	f_{NMR}	f_{DFT}	f_{XRD}	$r_{\mathrm{eff}}^{\mathrm{NMR}}(r_{\mathrm{eff}}^{\mathrm{DFT}})$ (pm)	Δr^{DFT} (pm)	$r_{\mathrm{eff}}^{\mathrm{NMR}}(r_{\mathrm{eff}}^{\mathrm{XRD}})$ (pm)	Δr^{XRD} (pm)
Pser							
**C**OOH–COO**H**	0.075	0.010	0.021	139(194)	−55	128(158)	−30
**C**H$_2$–C**H**^{R}H^{S}	0.239	0.316	0.121	115(110)	5	105(118)	−13
**C**H$_2$–CH^{R}**H**^{S}	0.191	0.311	0.238	119(110)	9	109(105)	4
**C**H–N**H**$_3$	0.047	0.020	0.020	181(209)	−28	166(191)	−25
**C**H–C**H**	0.428	0.312	0.577	104(110)	−6	96(91)	5
CaPser							
**C**OOH–C**H**	0.020	0.006	0.004	174(215)	−41	158(203)	−45
**C**H$_2$–C**H**^{R}H^{S}	0.322	0.309	0.280	109(110)	−1	99(102)	−3
**C**H$_2$–CH^{R}**H**^{S}	0.204	0.314	0.203	118(110)	8	107(107)	0
**C**H–N**H**$_3$	0.028	0.019	0.015	197(211)	−14	179(200)	−21
**C**H–C**H**	0.426	0.322	0.477	104(109)	−5	95(93)	2

[a] Effective ^{13}C–^{1}H distances (r_{eff}^{X}) and fractional dipolar contacts (f_X) for X={NMR, DFT, XRD}. Only ^{13}C–^{1}H interactions associated with f_X(C–H)$\geqslant$0.02 are listed. The uncertainties of the {f_{NMR}, f_{DFT}, f_{XRD}} data are {±15%, ±4%, ±4%}, whereas those of the corresponding effective distances are {±3%, ±0.7%, ±0.7%}. The respective correlation coefficients (R^2) between the {$r_{\mathrm{eff}}^{\mathrm{NMR}}$} distance set and those of {$r_{\mathrm{eff}}^{\mathrm{DFT}}$} and {$r_{\mathrm{eff}}^{\mathrm{XRD}}$} are 0.760 and 0.882 (Pser), and 0.914 and 0.911 (CaPser).

Table S5. 2Q and 1Q ^{1}H Chemical Shift Coordinates (in ppm)[a]

sites (δ_{H}^{j}/ppm)	COO**H**	PO**H**	N**H**$_3$	C**H**^{R}H	CH**H**^{S}	C**H**	**H**$_2$O
Pser							
COO**H**(16.6)	33.3	29.3; 12.6	24.9; 8.2	21.8; 5.1	20.7; 4.2	20.5; 3.9	
PO**H**(12.6)		25.3	20.9; 8.2	17.7; 5.1	16.8; 4.2	16.5; 3.9	
N**H**$_3$(8.2)			16.7	13.4; 5.1	12.3; 4.2	12.1; 3.9	
C**H**^{R}H(5.1)					9.3; 4.2	9.1; 3.9	
CH**H**^{S}(4.2)						8.0; 3.9	
CaPser							
N**H**$_3$(8.2)			16.4	13.0; 4.7	11.9; 3.7	*13.4; 5.2*	*13.4; 5.2*
C**H**^{R}H(4.7)					8.4; 3.7	9.9; 5.2	
CH**H**^{S}(3.7)						9.2; 5.2	
**H**$_2$O(5.2)							10.4

[a] Chemical shift coordinates observed in the 2Q–1Q ^{1}H NMR spectra of Fig. **7**. The 2Q shift ($\delta_{2\mathrm{Q}}$) of a proton pair of sites H^{j} (specified in the rows of the leftmost column) and H^{k} (columns at top) is given by $\delta_{2\mathrm{Q}} = \delta_{\mathrm{H}}^{j} + \delta_{\mathrm{H}}^{k}$, where slight deviations in the tabulated $\delta_{2\mathrm{Q}}$ values reflect experimental uncertainties. Each pair of numbers represent {$\delta_{2\mathrm{Q}}$, δ_{H}^{k}}, whereas each value of δ_{H}^{j} is specified within parentheses in the leftmost column. Only $\delta_{2\mathrm{Q}}$ is specified for the 2Q–1Q autocorrelations, for which $\delta_{\mathrm{H}}^{j} = \delta_{\mathrm{H}}^{k}$.

References

(S1) Mathew, R.; Pujari-Palmer, M.; Guo, H.; Yu, Y.; Stevensson, B.; Engqvist, H.; Edén, M. Solid-State NMR Rationalizes the Bone-Adhesive Properties of Serine- and Phosphoserine-Bearing Calcium Phosphate Cements by Unveiling Their Organic/Inorganic Interface. *J. Phys. Chem. C* **2020**, *124*, 21512–21531.

(S2) Edén, M.; Zhou, D.; Yu, J. Improved Double-Quantum NMR Correlation Spectroscopy of Dipolar-Coupled Quadrupolar Spins. *Chem. Phys. Lett.* **2006**, *431*, 397–403.

(S3) Teymoori, G.; Pahari, B.; Edén, M. Low-Power Broadband Homonuclear Dipolar Recoupling in MAS NMR by Two-Fold Symmetry Pulse Schemes for Magnetization Transfers and Double-Quantum Excitation. *J. Magn. Reson.* **2015**, *261*, 205–220.

(S4) Sundaralingam, M.; Putkey, F. F. Molecular Structures of Amino Acids and Peptides. II. A Redetermination of the Crystal Structure of L-*O*-Serine Phosphate. A Very Short Phosphate-Carboxyl Hydrogen Bond. *Acta Cryst.* **1970**, *B26*, 790–800.

(S5) Suga, T.; Okabe, N. Aqua(L-*O*-Serine Phosphato)Calcium(II). *Acta Cryst.* **1996**, *C52*, 1894–1896.
